# Visualizing the interplay of Cas1–Cas2 with DNA replication-repair that creates CRISPR–Cas immunity

**DOI:** 10.1093/nar/gkag564

**Published:** 2026-06-08

**Authors:** M Amin Hashemloo, Tom Killelea, Tomislav Mamić, Thomas H Ireland, Anna Lou-Hing, Fiona Kemm, Juachi U Dimude, Mirta Žagar, Ivana Ivančić-Baće, Christian J Rudolph, Edward L Bolt

**Affiliations:** Department of Life Sciences, Brunel University of London, Uxbridge, UB8 3PH, United Kingdom; School of Life Sciences, University of Nottingham, NG7 2UH, United Kingdom; Department of Molecular Biology, Faculty of Science, University of Zagreb, Horvatovac 102A, 10000 Zagreb, Croatia; School of Life Sciences, University of Nottingham, NG7 2UH, United Kingdom; School of Life Sciences, University of Nottingham, NG7 2UH, United Kingdom; School of Life Sciences, University of Nottingham, NG7 2UH, United Kingdom; Department of Life Sciences, Brunel University of London, Uxbridge, UB8 3PH, United Kingdom; Department of Molecular Biology, Faculty of Science, University of Zagreb, Horvatovac 102A, 10000 Zagreb, Croatia; Department of Molecular Biology, Faculty of Science, University of Zagreb, Horvatovac 102A, 10000 Zagreb, Croatia; Department of Life Sciences, Brunel University of London, Uxbridge, UB8 3PH, United Kingdom; School of Life Sciences, University of Nottingham, NG7 2UH, United Kingdom

## Abstract

Prokaryotic CRISPR–Cas systems rely on the Cas1–Cas2 protein complex to capture new DNA from mobile genetic elements (MGEs), to form immunological memory that defends against the MGEs. However, the mechanisms by which Cas1–Cas2 locates suitable DNA substrates inside cells remain unclear, limiting our understanding of how CRISPR–Cas immunity arises *de novo*. We directly visualized functional, DNA-bound Cas1–Cas2 complexes in bacteria, revealing the processes that license Cas1–Cas2 to capture DNA. Visible DNA-bound Cas1–Cas2 complexes formed only when replisomes are actively advancing, accumulating at post-replicative DNA gaps behind replication forks—structures arising during normal genome duplication, which are normally repaired by homologous recombination. Replication stress, which increases replicative DNA gap frequency, enhanced visible Cas1–Cas2 DNA binding. DNA capture by Cas1–Cas2 was strongly stimulated in cells lacking the RecFOR complex, which normally directs DNA gaps to repair. The RecBCD recombination initiator complex was essential for DNA capture by Cas1–Cas2 in these cells. The findings support a model in which naïve CRISPR–Cas adaptation is licensed by abundant replication-dependent DNA repair intermediates, prior to their repair by recombination. This identifies the mechanism co-ordinating Cas1–Cas2 with essential DNA replication and repair processes that all cells need, including when they are hijacked to replicate parasitic MGEs.

## Graphical Abstract

**Graphical Abstract:**
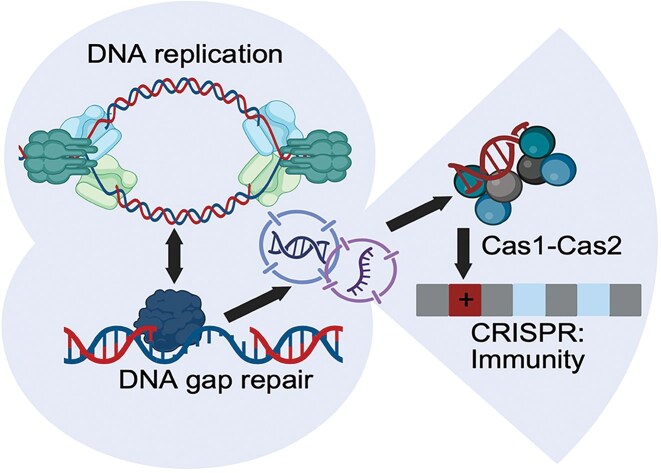

## Introduction

Bacteria and archaea edit their own genomes at specialized DNA sites formed of clustered regularly interspaced short palindromic repeats (CRISPRs). CRISPRs provide immunological ‘memory’ of cellular encounters with mobile genetic elements (MGEs), by gaining new ‘spacer’ DNA fragments from MGEs. CRISPRs are transcribed and processed into shorter CRISPR RNAs (crRNAs) that each comprise one spacer sequence, forming the basis of immune detection of returning MGEs [1]. *Cas* (CRISPR-associated) genes encode proteins that build the CRISPR DNA sites (adaptation) and utilize their crRNAs to provide a nucleolytic immune response that destroys the MGE (interference) [2]. Cas proteins are diverse in form, reflecting the ‘arms races’ between bacteria and archaea with MGEs [3]. However, the Cas1–Cas2 protein complex is found across CRISPR–Cas types, because it controls ‘adaptation’ to create CRISPR immunity.

Cas1–Cas2 protein complexes catalyse the capture of MGE DNA and its integration into CRISPR sites, which both creates and expands the CRISPR immune system [4–6]. When immunity is already established, from prior encounter with an MGE, Cas1–Cas2 locates MGE DNA through interaction with CRISPR interference protein complexes (e.g. Cascade-Cas3, Cas9). These complexes resect MGE DNA and provide DNA fragments for capture by Cas1–Cas2 [7–9], updating pre-existing immunity, in so called ‘primed adaptation’. However, when there is no pre-existing immunity, and therefore interference complexes are impotent, Cas1–Cas2 creates immunity *de novo* by capturing MGE DNA, so called ‘naïve adaptation’. Naïve adaptation needs active DNA replication [10, 11] linked with DNA repair [10, 12–14]. Cas1–Cas2 binds with high affinity to single-stranded or gapped DNA, as well as the duplex DNA of a capture complex, and can anneal complementary single-stranded DNA (ssDNA) into duplexes for integration into CRISPR sites [4, 15, 16], and Cas1 is active as a nuclease [13, 14, 17–19].

DNA gaps arise in chromosomes during genome duplication, and must be filled to ensure that genome duplication is completed. They form in the wake of advancing replisomes that have encountered DNA damage that prevents DNA synthesis but can be ‘skipped’ [20–22]—in *Escherichia coli* during normal genome duplication >1% (∼50 000 nucleotides) of the genome is gapped, rising to 5% after ultraviolet (UV) irradiation of cells [23]. Gap repair is achieved by homologous recombination [24, 25], often called ‘post-replication DNA gap repair’ or ‘template switching’ [26–29]. In bacteria, recombination is controlled by protein complexes RecBCD, a helicase-nuclease, and RecFOR, a DNA- and protein-binding hub complex. Each can load RecA recombinase onto SSB-bound ssDNA, thereby initiating DNA strand exchange that primes polymerases to fill the gaps [26, 30–37]. The importance and distinct roles of RecBCD and RecFOR in supporting DNA replication is clear from reduced viability of *E. coli* cells undergoing UV-induced replication stress when lacking a single complex, made worse in cells lacking both [37–40].

Cas1–Cas2 requires RecBCD for DNA capture into CRISPR sites [10, 12, 13] but has no requirement for the RecA-recombination reaction [12, 13]. It is not known whether RecFOR is involved, or how genome duplication and DNA repair license Cas1–Cas2 to capture DNA. By directly visualizing the dynamics of Cas1–Cas2 complexes in combination with replisomes and chromosomes in *E. coli* cells, and by measuring DNA capture by Cas1–Cas2 into their CRISPR-1 locus when cells are normally growing or stressed, we identified that targeting of replicative DNA gaps by Cas1–Cas2 evolves CRISPR-1 by adding new DNA. It highlights that it might in fact be parasitic MGE genome replication that is leveraged for DNA fragments that provide cellular CRISPR–Cas immunity. The dynamic behaviour of Cas1–Cas2 in response to endogenous and exogenous DNA stressors also reveals its potential as a novel biomarker for DNA replication coupled repair.

## Materials and methods

### Strains, plasmids, media, and general methods

*Escherichia coli* strains and plasmids are listed in the Supplementary Data Tables. *Escherichia coli* cells used for spacer integration assays lack chromosomal *cas1* and *cas2* genes but have the chromosomal CRISPR-1 site. Therefore we assess DNA capture into CRISPR-1 arising from ectopic expression of Cas1-eYFP-Cas2 and its mutants. The *recF*^−^, *recO*^−^*, recR^−^*, and *recB^−^* bacterial strains, and their derivatives, were made by P1*vir* transduction and selected for the appropriate antibiotic resistance or by recombineering [41] to insert kanamycin resistance cassette followed by P1 *vir* transduction into strain BW25113 or MG1655, as described in detail in Ivancic-Bace *et al*. [42]. Bacteria were grown at 37°C in LB broth (10 g/l bacto-tryptone, 5 g/l yeast extract, 10 g/l NaCl) and on LB agar plates (supplemented with 15 g/l of agar for solid media).

### DNA and proteins and their analysis *in vitro*

DNA oligonucleotides used for polymerase chain reaction (PCR) and *in vitro* protein assays are listed in Supplementary Table S3. Cas1 and Cas1^V76L^ and their Cas1–Cas2 complexes were purified using the same procedures, described in Killelea *et al*. [11]. For EMSAs the Cas1 proteins were diluted to working concentrations in 20 mM Tris (pH 8.0), 100 mM NaCl, and 1 mM DTT, prior to mixing with DNA. Cas1 proteins were incubated at room temperature for 5 min with 50 nM of Cy5 end-labelled ssDNA tailed duplex (Supplementary Data) in 20 mM Tris (pH 8.0), 0.1 mg/ml bovine serum albumin (BSA), and 7% glycerol. Reactions were loaded onto a 5% native acrylamide gel and migrated at room temperature for 90 min at 5 W. For disintegration assays the same reactions as for EMSAs were instead incubated for 30 min at 37°C in buffer supplemented with 10 mM MgCl_2_, with reactions ended by addition of 1 µl Stop Buffer (0.2 mg/ml proteinase K, 2% sodium dodecyl sulphtate, and 100 mM ethylenediaminetetraacetic acid) and 42.5% (w/v) formamide and bromophenol blue loading dye. These reactions were heated to 95°C immediately prior to loading onto a 12% (w/v) urea gel in TBE buffer. Gels were imaged directly on a Typhoon™ laser scanner platform and viewed in ImageJ.

DNA fluorescence anisotropy measurements used Cas1 proteins as shown in the ‘Results’ section, after dilution in buffer (20 mM Tris.HCl pH 8.0, 100 mg/mL BSA, 7% v/v glycerol). Anisotropy reactions were done in triplicate, maintained at 37°C for measurements of fluorescence polarization in a FLUOstar Omega Microplate Reader (BMG Labtech) after 10 min of incubation. The measurements were plotted using GraphPad Prism and analysed using nonlinear regression, the one-site total binding model. Total binding was used over specific binding because although we had pre-optimized substrate binding by Cas1–Cas2 (Supplementary Fig. S4), Cas1, and Cas1–Cas2 complexes could potentially still bind to any area of the model substrate, via non-specific interactions. Further explanation and the equation for this curve fit is detailed in https://www.graphpad.com/guides/prism/10/curve-fitting/reg_one_site_total.htm.

### Single-image microscopy of fluorescently-tagged proteins in *E. coli* cells

The Cas1-eYFP complex was generated as described in Killelea *et al*. [11]. For cell imaging, fresh overnight cultures of the *E. coli* strains of interest were diluted 100-fold in fresh LB broth (Miller composition) and incubated with vigorous aeration at 37°C until OD_600_ reached 0.2. Ampicillin was added to the culture at a final concentration of 50 µg/ml to maintain plasmid selection. At OD_600_ 0.2, expression of Cas1–Cas2 protein complex from the pBad plasmid was induced by adding l-arabinose [0.1% (w/v)] and cells were incubated for further 60 min. If included, saccharin was added at the same time as l-arabinose to the final concentration indicated in the figures. For staining of the nucleoid, Hoechst 33342 (Thermo Scientific™) was added to a taken sample to a final concentration of 10 µg/ml and incubated for 5 min at room temperature.

For visualization of yellow fluorescence, 1 µl sample was directly pipetted onto an agarose pad and air-dried. For generation of pads, a 65-µl (15 × 16 mm) GeneFrame (Thermo Fisher Scientific) was added to a conventional microscopy slide. One percent of SeaKem LE Agarose (Lonza) was added to 1× M9 minimal medium (diluted from a 5× stock) and heated until the agarose was completely dissolved. This solution (95 µl) was added into the GeneFrame chamber and the chamber sealed immediately with a conventional microscopy slide. Once set, the top slide was removed and the agarose pad air-dried for a maximum of 5 min at 42°C and used immediately. Once the sample was added and air-dried, the GeneFrame chamber was sealed by adding a 22 × 22 mm cover slip. Visualization of foci used a T*i*-U inverted microscope (Nikon) with a CFI Plan Fluor DLL 100× objective (Nikon) and an ORCA Flash 4.0 LT plus camera (Hamamatsu). Phase contrast images were taken using a pE-100 single LED wavelength source (CoolLED). For fluorescence, the pE-4000 illumination system (CoolLED) was used. The relevant filters for visualization of YFP was Zeiss filter set 46. Hoechst 33342 was captured via a DAPI-50LP-A filter, If red fluorescence was imaged in addition to YFP, following arabinose induction of expression cultures were washed with and resuspended in 1× M9 minimal medium for the purpose of reducing background fluorescence caused by the aromatic compounds in rich medium. Culture (1 µl) was transferred onto an agarose pad, as described above. Visualization was done with the same microscope, but with a Plan Apo 100× Lambda objective (Nikon), using the Nikon filter TXRED-A-Basic (mCherry). Images were captured using the NIS Elements-BR software V4.51 (Nikon). Standard exposure settings were 100 ms at 50% intensity for Hoechst 33342, 1 s at 75% intensity for Cas1-linker-eYFP-Cas2, and 3 s at 75% intensity for mCherry-LacI. Postprocessing, such as cropping and rotating, was performed in Adobe Photoshop CC (V26.7). For all single image microscopy experiments a minimum of two biological replicates were performed, with a minimum of three individual frames being recorded per condition and experiment. For foci numbers, cells were counted from two separate experiments, using a minimum of three individual frames per experiment. The total number of cells counted is stated in the text or in the figures.

### Analysis of foci brightness in cells

Semi-quantitative analysis of bright Cas1–Cas2 foci in cells used a different analysis pipeline. Images were processed using Fiji (ImageJ Version 1.54p). The brightness of the fluorescence channel of an image from *recF*^−^ cells was manually adjusted so that the brighter foci were clearly identifiable. The numerical adjustment values of the change in brightness were then copied across both to further images of *recF*^−^ and wild-type cells, to ensure precisely the same brightness adjustment was made for all images analysed. The brightness-adjusted fluorescence channels were then transformed in Fiji to a 16-colour heat map, ranging from blue to white. In data from two replicate experiments, with three frames per experiment, it was then counted how many cells were visible and how many of those counted cells showed foci reaching all the way to ‘white’ in the heat map. The statistical analysis was performed in MS Excel (Version 2108) as a two tailed *z*-test.

### Single-image microscopy using temperature-sensitive alleles

For visualization of Cas1–Cas2 in strains carrying the temperature-sensitive alleles *dnaA46, dnaC7*, and *dnaE486*, cells were grown at the permissive temperature of 30°C to an OD_600_ of 0.2. Aliquots (1 ml) were then transferred into pre-warmed glass tubes and incubated either at 30°C or the restrictive temperature of 42°C. In our experiments we observed that expression and maturation of fluorescently-tagged proteins did not produce reliable signal following the 60-min induction period we used for cultures grown at 37°C. We therefore imaged cells grown at 30°C following a 120-min induction period. For the restrictive temperature of 42°C, we took samples after 90 min to allow for replication run-out, or after 90 and 120 min, as indicated in the Figures. Visualization was otherwise done as described above.

### Single-image microscopy after UV irradiation

UV irradiation methods are derived from prior analysis of *E. coli* DNA repair phenotypes [43, 44]. Fresh overnight cultures of the *E. coli* strains were diluted 100-fold in fresh LB broth and incubated with vigorous aeration at 37°C until OD_600_ reached 0.2. Ampicillin was added to the culture at a final concentration of 50 µg/ml to maintain plasmid selection. At OD_600_ 0.2, Cas1–Cas2 expression was induced by adding l-arabinose [0.1% (w/v)]. The cells were then incubated for 30 min to start transcription of Cas1–Cas2 before any UV-induced damage occurs. To avoid any absorption of UV by the medium used, cells were spun down, resuspended in 100 µl of the supernatant, and then plated on the surface of an LB agar plate. Once the surface of the plate was dry, the surface was irradiated with a 254-nm germicidal lamp (4 W; Farnell UK), adjusted to an output of 1 W/m^2^. The remaining supernatant was sterile-filtered to remove any cells that were not irradiated. The filtered supernatant was then used to wash the surface of the irradiated plate to recover all cells. These cultures were then incubated at 37°C and samples were taken at the times indicated. For UV irradiation of cells with DnaN-YPet, neither ampicillin nor arabinose were needed, as the marker is chromosomally integrated and expressed from its native promoter. However, otherwise the above procedure was followed so that the experimental conditions were matched as closely as possible. Visualization was done using the T*i*-U inverted microscope (Nikon), as described above.

### Fluorescent repressor-operator system microscopy

Strain JD1841(Supplementary Table S2) was used for fluorescent labelling of *oriC*/*dif* regions. This was generated by transforming RRL189 (Supplementary Table S2) with pTK135 (Supplementary Table S4) to allow simultaneous visualization of *oriC, dif*, and Cas1–Cas2. JD1841 carries a *lacO_240_* DNA sequence array located 16 kb anticlockwise from *oriC* and a *tetO_240_* DNA sequence array 50 kb clockwise from the chromosomal dimer resolution site *dif*. The sites were illuminated by constitutively expressing the LacI repressor fused to mCherry and the TetR repressor fused to mCerulean from two chromosomal integration locations. Cas1–Cas2 was expressed from the expression plasmid, as described above.

### DNA capture (naïve adaptation) assays and DNA sequence analysis

DNA capture into the chromosomal CRISPR-1 sites was assessed from arabinose inducible co-expression of Cas1 and Cas2 in the plasmid vector pBad-HisA (Invitrogen), alongside an empty vector negative control, described in detail in Ivancic-Bace *et al*. [12]. Overnight cultures of cells transformed with these plasmids containing ampicillin (50 μg/ml) and 0.2% (w/v) l-arabinose were grown from a single colony. This is ‘Passage 1’ (P1). Dilution (1:300) of P1 into fresh media containing 0.2% (w/v) l-arabinose was then grown with shaking in a baffled flask to OD_600_ of 0.6, and arabine added again to 0.2% (w/v) and allowed to continue growing for 8 h, generating ‘Passage 2’ (P2). Samples of growing P1 and P2 (100 µl) were mixed with SDW (400 µl) and heated at 95°C for 10 min, then frozen and thawed for PCR to detect spacer acquisition. The primer pairs used for PCR detection and strains are listed in the Supplementary Tables. Spacer acquisition was measured as the percent of the total DNA that had been expanded in the CRISPR locus, as indicated by arrows in the gel panels.

For high-throughput sequencing analysis of spacers the naïve adaptation assays were done through three passages for wild-type strain IIB1165 and twice for *recF*^−^ strain IIB1467, each as two independent replicates. PCR products of the extended CRISPR-1 array were gel purified using the Thermo Scientific GeneJET PCR Purification Kit. The primers used for CRISPR amplification were CRISPR-NGS-F 5′-TGCTTTAAGAACAAATGTATACTTT-3′ and CRISPR-NGS-R 5′-CAACATTATCAATTACAACCGA-3′.

Next-generation sequencing (NGS) was performed on the NovaSeq X platform using a 2 × 150 bp paired-end configuration. DNA quality control, library preparation, and bioinformatics were by Macrogen-Europe. Raw sequencing reads were pre-processed by trimming adapter sequences with Trimmomatic v0.38. Overlapping paired-end reads were then merged using FLASH v1.2.11 and subsequently quality-filtered with PRINSEQ-lite v0.20.4. The processed sequences were used for downstream CRISPR-spacer analysis.

Template size distribution was assessed using the Agilent 2100 Bioanalyzer, and library quantification was performed according to the Illumina qPCR Quantification Protocol Guide [TruSeq Nano DNA (LMW) library].

Spacer identification and extraction were performed using an in-house script. Reads containing four occurrences of the CRISPR repeat sequence (≤2 mismatches, no gaps) were identified and classified as ‘extended CRISPR + 61 bp’. From these reads, the spacer between the first and second repeat sequences was extracted and spacer sequences were filtered by length (31–33 bp) according to analysis guidelines.

The R packages ShortRead and Biostrings were used for DNA read pre-processing and downstream analysis, including mapping and visualization. Spacer sequences of 31–33 bp in length were extracted from the extended reads between CRISPR repeats. Spacer mapping was performed in two steps: first, spacers were aligned to the plasmid sequence, retaining only uniquely mapped reads. Spacers that did not match to the plasmid were then aligned to the *E. coli* K-12 genome (NC_000913.2). Non-unique matches were excluded. Up to 1–2 mismatches were allowed when aligning to plasmid and chromosome sequences. Mapping was performed using BLASTn with a stringent *e*-value threshold (−e 0.0001) and the Bowtie 2 aligner. Spacers aligned to repetitive rRNA regions or multiple genomic locations were discarded. For the statistical analysis of the spacer distribution, each unique spacer was counted only once, regardless of its abundance. The MarkDuplicates (Picard) tool was used to remove redundant spacer reads. Spacer distribution was visualized using 10 kb genomic bins. The data of the biological replicates were merged before visualization.

### Bimolecular fluorescence complementation

For bimolecular fluorescence complementation (BiFc) we used the ‘split mVenus’ method [45] measuring fluorescence from within *E. coli* cells co-expressing protein fusions; N-mVenus-Cas1 and C-mVenus DnaK. Plasmids (listed in Supplementary Tables) were transformed into BL21AI cells and overnight cultures were used to inoculate 2 ml of LB broth supplemented with ampicillin at 100 μg/ml and kanamycin at 50 μg/ml to select for both plasmids, within a 24-well plate. Cultures were incubated at 37°C with shaking at 200 rpm in a FLUOstar Omega Microplate Reader (BMG Labtech). At OD_600_ of 0.6, cells were treated with l-arabinose [0.2% (w/v)] and IPTG (1 mM) to induce Cas1, Cas2, and DnaK protein expression. After four hours 1 ml of cell culture was harvested and resuspended in 200 μl of 1× M9 salts. Relative fluorescent intensity was measured in a FLUOstar Omega Microplate Reader (BMG Labtech) with excitation and emissions filters set at 500 nm and 520 nm, respectively.

## Results

### Cas1 alone is not sufficient for Cas1-eYFP focus formation in cells—it needs to form stable Cas1–Cas2 complexes

**Figure 1.**
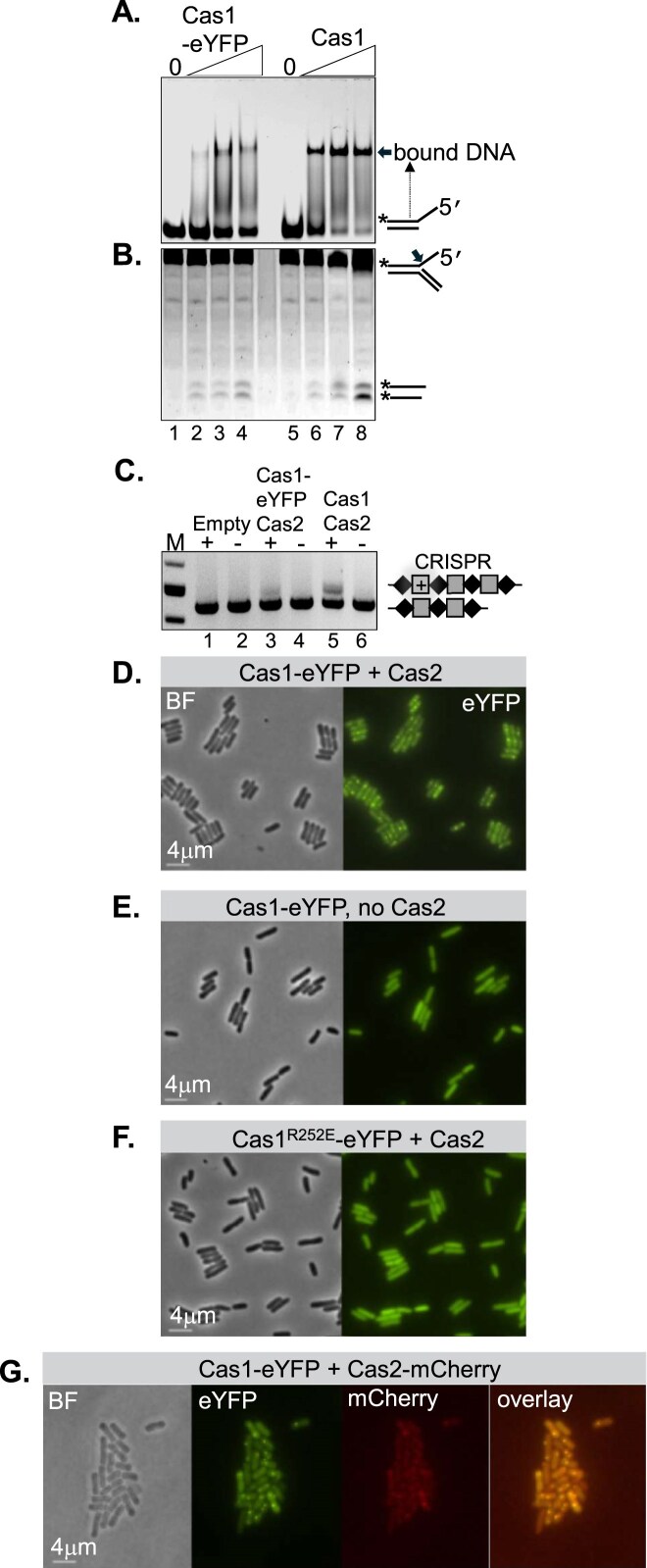
Cas1-eYFP focus formation in *E. coli* cells requires formation of stable Cas1–Cas2 complex. (**A**) EMSA showing binding of purified Cas1-eYFP (lanes 1–4, Supplementary Fig. S1) and Cas1 (lanes 5–8) (250, 500, and 750 nM) to Cy5 end-labelled tailed duplex DNA (50 nM), forming a stable protein–DNA complex in each case, with no sign of abnormal protein aggregates in the gel wells. (**B**) Reaction products in a denaturing urea gel from reactions to test catalysis by Cas1-eYFP (lanes 1–4) compared with Cas1 (lanes 5–8) (250, 500, 750 nM), on a cy5 end-labelled forked DNA substrate (50 nM). The assay reconstitutes Cas1 DNA cutting ‘disintegration’ activities shown previously *in vitro* [14, 46]. (**C**) New DNA capture into the *E. coli* chromosomal CRISPR-1 locus detected by PCR—naïve adaptation. The agarose gel shows PCR products from expansion of CRISPR-1, after capture and integration of DNA fragments. Lanes 1 and 2 show no new DNA capture from an empty plasmid vector when induced (+) or not (−), compared with a plasmid expressing Cas1-eYFP + Cas2 (lanes 3 and 4) or Cas1 + Cas2 (lanes 5 and 6), in *E. coli* cells lacking chromosomal *cas1* and *cas2* genes. (**D)** Each microscopy panel (in parts D–G) shows brightfield (BF) and fluorescent (eYFP, mCherry) images. *Escherichia coli* MG1655 cells co-expressing Cas1-eYFP and Cas2 from a single plasmid show foci when grown in rich media. (**E)** Elimination of Cas2 from the co-expression plasmid, in otherwise identical conditions, eliminates all foci. (**F)** Substituting Cas1-eYFP for Cas1^R252E^-eYFP—that is unable to form stable Cas1–Cas2 complex [5]—and co-expression of Cas2 eliminates foci in otherwise identical conditions. (**G**) Overlapping Cas1-eYFP and Cas2-mCherry foci in cells as further evidence for association of Cas1-eYFP and Cas2 in stable complexes.

To visualize Cas1–Cas2 in cells, we fused *E. coli* Cas1 protein to enhance yellow fluorescent protein *via* a (Gly-Gly-Ser)_8_ linker, and showed that the purified protein (Cas1-eYFP, Supplementary Fig. S1) binds and cuts DNA *in vitro*, as expected for active Cas1 (Fig. 1A and B), albeit with reduced efficacy compared with Cas1. Co-expressing Cas1-eYFP with Cas2 from a plasmid in *E. coli* cells that lack chromosomal *cas1* and *cas2* showed DNA capture into the chromosomal CRISPR locus (naïve adaptation), compared with a plasmid vector-only control (Fig. 1C). Cas1-eYFP is therefore catalytically active *in vitro* and, with Cas2, captures DNA into the CRISPR site *in vivo*.

Cas1-eYFP foci were readily observable in cells expressing Cas1-eYFP and Cas2 when growing in rich media, with most cells showing one (22%), two (38%), or three (18%) foci *per* cell (Fig. 1D). These foci disappeared entirely when the *cas2* gene was deleted from the plasmid (Fig. 1E), suggesting that Cas1-eYFP foci represent only Cas1–Cas2 complexes, rather than Cas1 oligomers that have been observed *in vitro* [47]. Further support for this was obtained by substituting Cas1-eYFP for Cas1^R252E^-eYFP, a mutation abolishing an electrostatic interaction required to form a stable Cas1–Cas2 complex, which therefore cannot capture DNA into CRISPR in cells [5]—these cells also lacked Cas1-eYFP foci (Fig. 1F). Expressing a Cas1-eYFP-Cas2-mCherry complex—that was also proficient at DNA capture into CRISPR (Supplementary Fig. S2)—gave co-localized eYFP and mCherry foci (Fig. 1G). However, in some cells Cas2-mCherry foci were not visible at all, presumably due to the lower stoichiometry of Cas2 in the active (Cas1)_4_–(Cas2)_2_ complexes, and because mCherry fluorescence is less brighter than eYFP fluorescence. Therefore, we instead created Cas1-mCherry and Cas2-eYFP fusions to improve focus resolution, but this combination did not support DNA capture into CRISPR in cells (Supplementary Fig. S2A).

In summary, the fact that Cas1-eYFP foci are absent if Cas2 is absent or cannot interact with Cas1, plus overlapping Cas1-eYFP and Cas2-mCherry foci in many cells, indicates that foci in these cells represents functional Cas1–Cas2 complexes.

### Cas1–Cas2 mutations alter behaviour of Cas1–Cas2 foci

**Figure 2.**
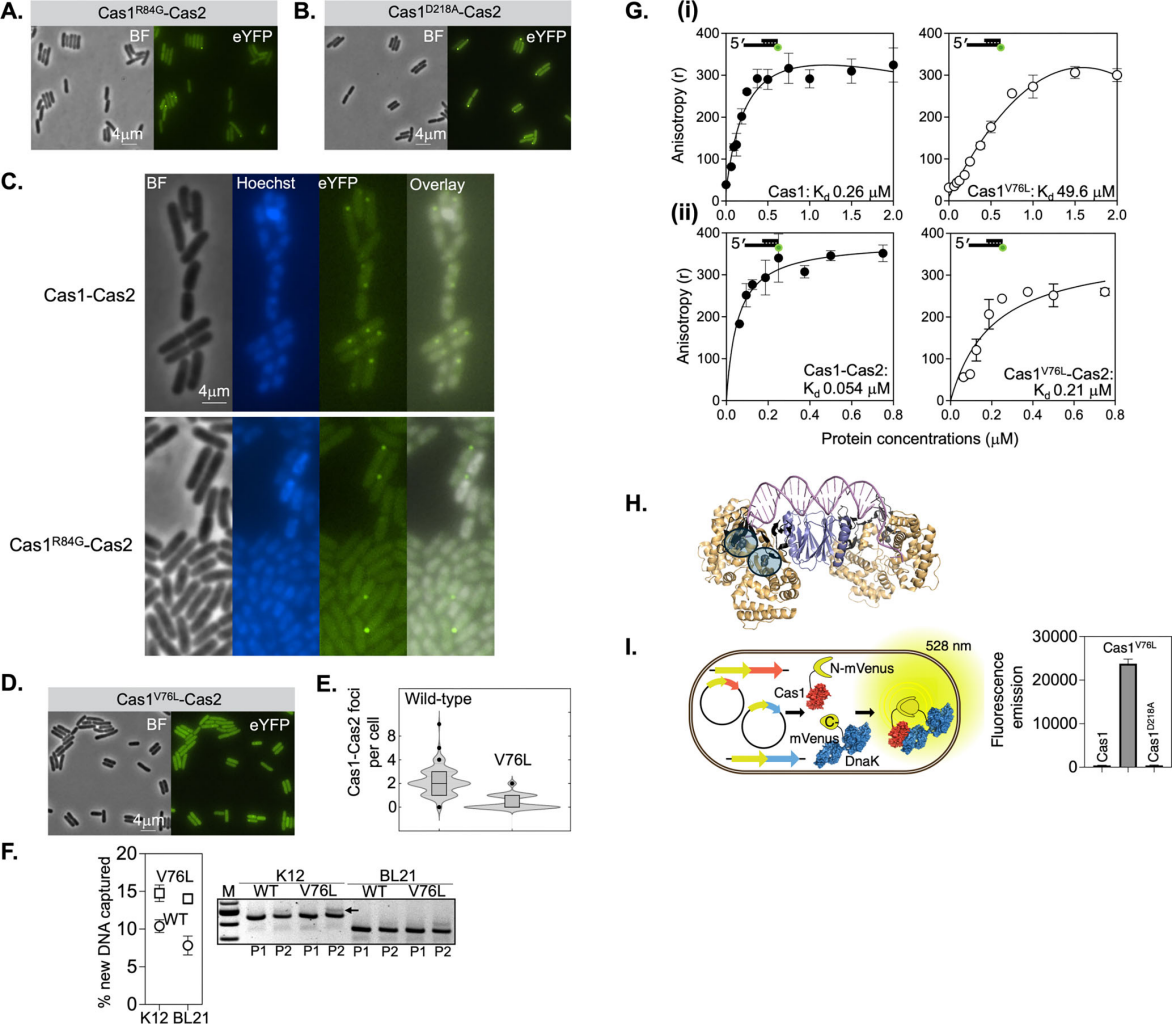
Cas1–Cas2 focus dynamics of mutant complexes. (**A, B**) Brightfield (BF) and fluorescent (eYFP) imaging of cells with absent or polar cellular foci from expression of Cas1^R84G^–Cas2 or Cas1^D218A^–Cas2. (**C)** Microscopy images of live cells expressing Cas1–Cas2 or Cas1^R84G^–Cas2, shown as brightfield (BF) and fluorescence (Hoechst 33342 DNA stain and eYFP). In the merged Hoechst and eYFP panels the Hoechst DNA stain is shown as white, to be able to resolve the yellow of eYFP. Cas1–Cas2 foci are all present within the white area of the merge, consistent with the nucleoid, but Cas1^R84G^–Cas2 foci are clearly outside of it. (**D**) Fewer, less bright, foci from Cas1^V76L^–Cas2 compared with Cas1–Cas2. (**E**) Combined violin-box plots of Cas1^V76L^–Cas2 or Cas1–Cas2 focus distribution, in each case from 750 cells in total. Dots on the plot represent outlier counts. The box inside the violin represents 75% of the total data points and shows the median data point as a line; two foci for Cas1–Cas2 and zero for Cas1^V76L^–Cas2. (**F**) DNA capture into CRISPR-1 from expressing Cas1^V76L^–Cas2 (squares) compared with wild-type Cas1–Cas2 (WT, circles), confirming prior observations made in different *E. coli* strains [50]. Measurements were in *E. coli* K-12—for observing Cas1–Cas2 foci—and *E. coli* BL21—for DNA capture assays. The data are mean values with standard error shown from three independent experiments, each measuring expanded CRISPR loci after two passages of growth. On the right, a representative gel illustrates the hyper-active DNA capture into CRISPR-1 by Cas1^V76L^–Cas2, compared with Cas1–Cas2. The arrows points to PCR DNA products resulting from new DNA integration into CRISPR-1, after one (P1) or two (P2) growth passages. (**G**) DNA binding changes induced by the Cas1^V76L^ mutation. The graphs show DNA binding measured as fluorescence anisotropy from an optimized DNA substrate (40 nM) for Cas1–Cas2 (see Supplementary Fig. S4). Proteins were used at the following (nM) concentrations: 62.5, 95, 125, 187, 250, 375, 500, 750, 1000, 1500, and 2000 for Cas1 only, and the same concentrations up to 750 nM for the Cas1–Cas2 complexes, which could not be isolated at as high concentrations as Cas1 alone. Each titration measured DNA anisotropy over 50 min, generating binding data from three independent reaction wells as indicated in the graphs with mean and standard error, and described in the ‘Materials and methods’ section. Nonlinear regression for each curve was used to generate ‘best fits’ and a Kd value for each, as is given on the graph. (**H**) *Escherichia coli* (Cas1)_4_-(Cas2)_2_ DNA ‘capture’ complex (PDB 5DS4 [4]) with four Cas1 monomers in gold, two Cas2 monomers in purple, and DNA in pink. Highlighted in black within each Cas1 monomer is the beta-sheet region (amino acids 21–60) predicted to interact physically with DnaK (Supplementary Fig. S6). Valine-76 is circled to the left, in two of the Cas1 monomers, located directly underneath the beta-sheet region. (**I**) Cas1 physically interacts with DnaK [11], measured using BiFC when co-expressing DnaK and Cas1 proteins within cells and recording fluorescence emission at 528 nm. Fluorescence emission from expressing NmVenus-Cas1^V76L^ with DnaK-CmVenus was many thousands of times stronger than NmVenus-Cas1. The data are means from three independent experiments showing bars for standard error.

We investigated the effect on foci of mutations that inactivate DNA processing by Cas1–Cas2. Cas1^R84G^–Cas2 and Cas1^D218A^–Cas2 are unable to capture DNA into CRISPR sites in cells, because of defective DNA binding and integration reactions [4, 11, 12]. When these mutations were introduced into Cas1-eYFP–Cas2 we observed that most cells lacked any foci, or in 10%–20% of cells there was a single focus only at one cell pole, rather than the typically 1–3 foci located centrally (Fig. 2A and B, compared with Fig. 1D). The same effect on foci was observed when imaging a prototype Cas1-eYFP-Cas2 complex that we had made, but which was inactive at DNA capture and integration because it lacked the (GGS)_8_ peptide linker (Supplementary Fig. S2B). This polar compartmentalization of proteins in *E. coli* indicates their dysfunctionality [48, 49], consistent with inactive mutant Cas1–Cas2 complexes. When Cas1–Cas2 or Cas1^R84G^–Cas2 foci were imaged in conjunction with Hoechst 33342 DNA stain, we observed most Cas1–Cas2 foci within the nucleoid region of cells, but the rare Cas1^R84G^–Cas2 foci were at cell peripheries, with no obvious overlap with nucleoid DNA (Fig. 2C). The data support that foci represent Cas1–Cas2 complexes engaging with cellular DNA.

We also assessed the mutant complex Cas1^V76L^–Cas2, which gave many cells with no foci or cells with foci of reduced intensity (Fig. 2D and E). This was surprising because Cas1^V76L^–Cas2 is hyper-active at DNA capture in cells [50], prior work confirmed in the *E. coli* strains we used (Fig. 2F). The presence of some foci from this mutant suggested that unstable DNA binding, in agreement with EMSA data [42], which we measured in real time using purified proteins (Supplementary Fig. S3) and fluorescence anisotropy of an optimized DNA substrate for Cas1 (ssDNA-tailed duplex—shown in Supplementary Fig. S4 and in Radovcic *et al*. and Kim *et al*. [13, 15]). Cas1^V76L^ gave 200-fold reduced affinity of DNA binding compared with Cas1 (K_d_ 49.6 μM for Cas1^V76L^and 0.26 μM for Cas1, Fig. 2G-i). DNA binding affinity of Cas1^V76L^ was much improved in a Cas1^V76L^–Cas2 complex (K_d_ 0.21 μM) but this was four-fold lower affinity binding to DNA than Cas1–Cas2 (K_d_ 0.054 μM), measured in triplicate as a function of time (Fig. 2G-ii). This magnitude of reduced DNA binding by Cas1^V76L^–Cas2 fits with diminished foci in cells, rather than their disappearance as observed with Cas1^R84G^–Cas2.

Valine-76 in *E. coli* Cas1–Cas2 abuts the Cas1 DNA binding sites within the Cas1–Cas2 complex (Fig. 2H), therefore its mutation probably perturbs DNA binding, as suggested by the data. But how could its disrupted DNA binding results in increased DNA capture? A clue arose from AlphaFold Multimer [51], which predicted that this region of Cas1 is the interaction site with the chaperone DnaK, which binds to Cas1 but not Cas2 *via* its substrate binding domain (Supplementary Fig. S5) [11]. We therefore measured physical interaction of Cas1 with DnaK in *E. coli* cells using BiFC [52] (Fig. 2I). Cas1^V76L^ and DnaK showed hugely enhanced physical interaction, compared with Cas1 and DnaK (Fig. 2H), and Cas1^V76L^ was unusually abundant in cells grown over several hours (Supplementary Fig. S6). Therefore, unstable DNA binding of Cas1^V76L^ may be overcome by its high stability and abundance during DNA capture assays that run over 12–18 h of bacterial growth.

Together, these Cas1–Cas2 mutants provide further support that Cas1–Cas2 foci represent stable active complexes that bind and capture DNA into CRISPR. We next directly visualized interaction of Cas1–Cas2 with DNA replication and DNA repair.

### Cas1–Cas2 binds DNA in response to active genome replication, stimulated by replication defects

**Figure 3.**
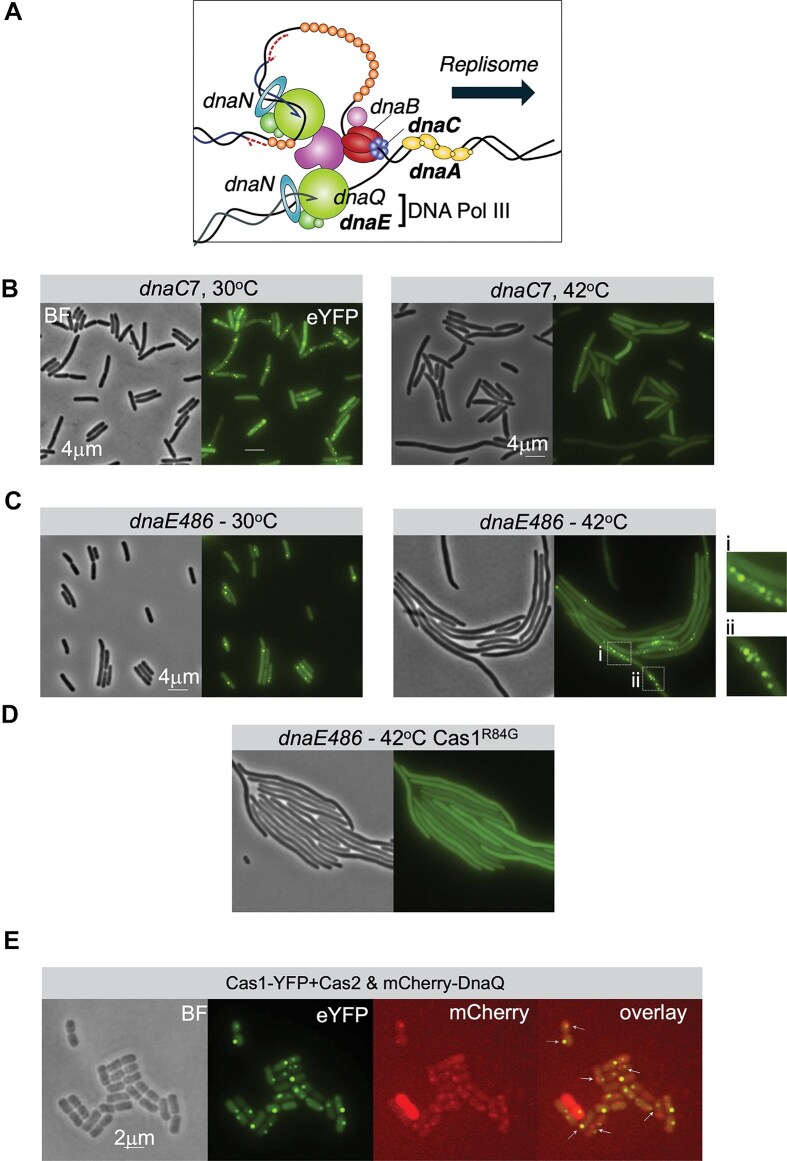
Cas1–Cas2 targets DNA structures behind advancing replication forks. (**A**) A scheme of the *E. coli* replisome, highlighting in bold text the replication protein mutants utilized, each described in detail in Lazowski *et al*. [53]. DnaA is also shown because it initiates DNA replication from origins. DnaQ and DnaE are protein subunits of replicative DNA polymerase III. DnaB is the replisome helicase complex that separates parental DNA ahead of polymerase III. DnaC is shown for its role at loading DnaB helicase during replication initiation, but during active extension of DNA synthesis by the replisome DnaC would have dissociated. DnaN is the ‘sliding clamp’ for DNA synthesis behind the advancing replisome, utilized in post-replicative DNA gap repair. (**B**) Each microscopy panel (B–G) shows brightfield (BF, left) and fluorescent (eYFP, right) images. Cas1–Cas2 foci in *dnaC7* cells at 30°C disappear at 42°C, when cells are unable to advance replisomes and form nascent DNA. (**C**) Cas1–Cas2 foci in *dnaE*486 cells replicating at 30°C become more intense and clustered in cells at 42°C, highlighted by inset boxes (i) and (ii), as cells lose replicative functionality of DNA polymerase III, inducing SOS replication stress. (**D**) Clusters of Cas1–Cas2 foci observed in filamented *dnaE*486 cells at 42°C after 120 min disappear if Cas1–Cas2 is substituted for Cas1^R84G^–Cas2, which is unable to bind to DNA [4, 12]. (**E**) Co-expression of Cas1-eYFP and Cas2 with replisome DnaQ-mCherry results in many distinct eYFP or mCherry foci in overlay (arrows), consistent with Cas1–Cas2 being away from the core replisome and DNA fork branchpoint.

**Figure 4.**
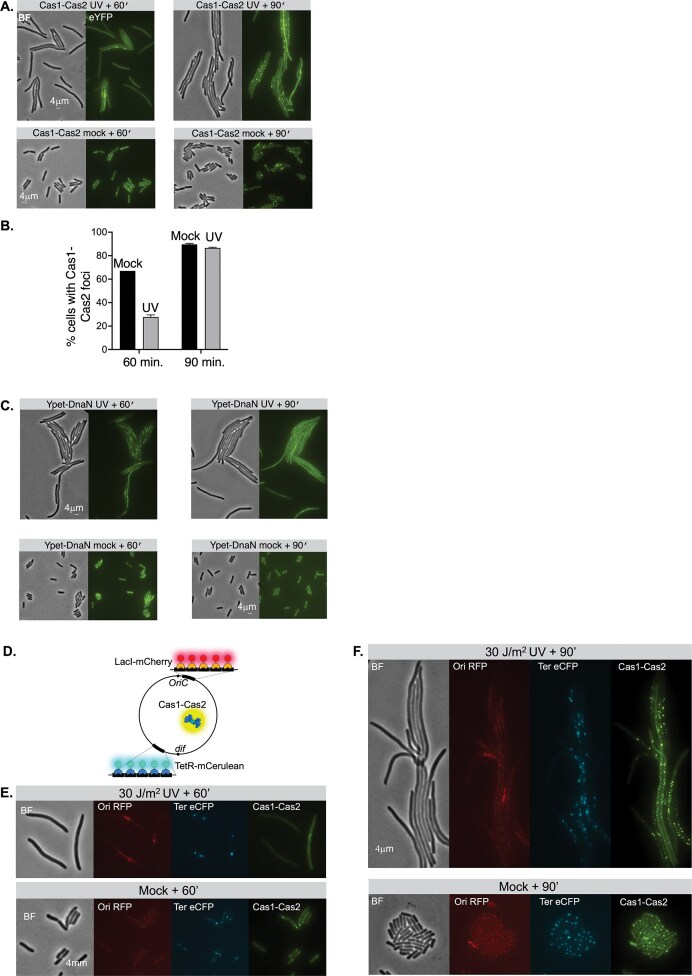
Dynamics of Cas1–Cas2, the replisome, and chromosomal sites in response to UV irradiation. Each microscopy panel shows brightfield (BF, left) and fluorescent (RFP, eCFP or eYFP, right) images. (**A**) Imaging of Cas1–Cas2 foci in cells either mock treated or treated by irradiation with 30 J/m^2^ of UV light with subsequent growth for the minutes (‘) indicated. (**B**) Counting cells containing Cas1–Cas2 foci, after mock treatment or irradiation with 30 J/m^2^ of UV light. Cells were grown post-treatment for 60 or 90 min as indicated, and cells were imaged and foci counted. For each treatment we counted 800 cells from two independent experiments. (**C)** Imaging cells for replisome (YPet-DnaN) foci. Cells were either mock treated or treated by irradiation with 30 J/m^2^ of UV light with subsequent growth for the minutes (‘) indicated. (**D**) FROS microscopy used in this work, combined with expression of Cas1–Cas2 complex. LacI-mCherry binds to *lacO*240 sequences close to the replication origin *oriC*, and TetR-mCerulean binds to *tetO*240 sequences close to *dif* in the replication termination region. (**E, F**) Imaging genomic sites TerA (eCFP-Ter, replisome termination) and OriC (RFP-Ori, Origin of replication) after mock treatment or UV irradiation as indicated, at the time points indicated. Supplementary Fig. S8 shows similar foci after 120 min.

**Figure 5.**
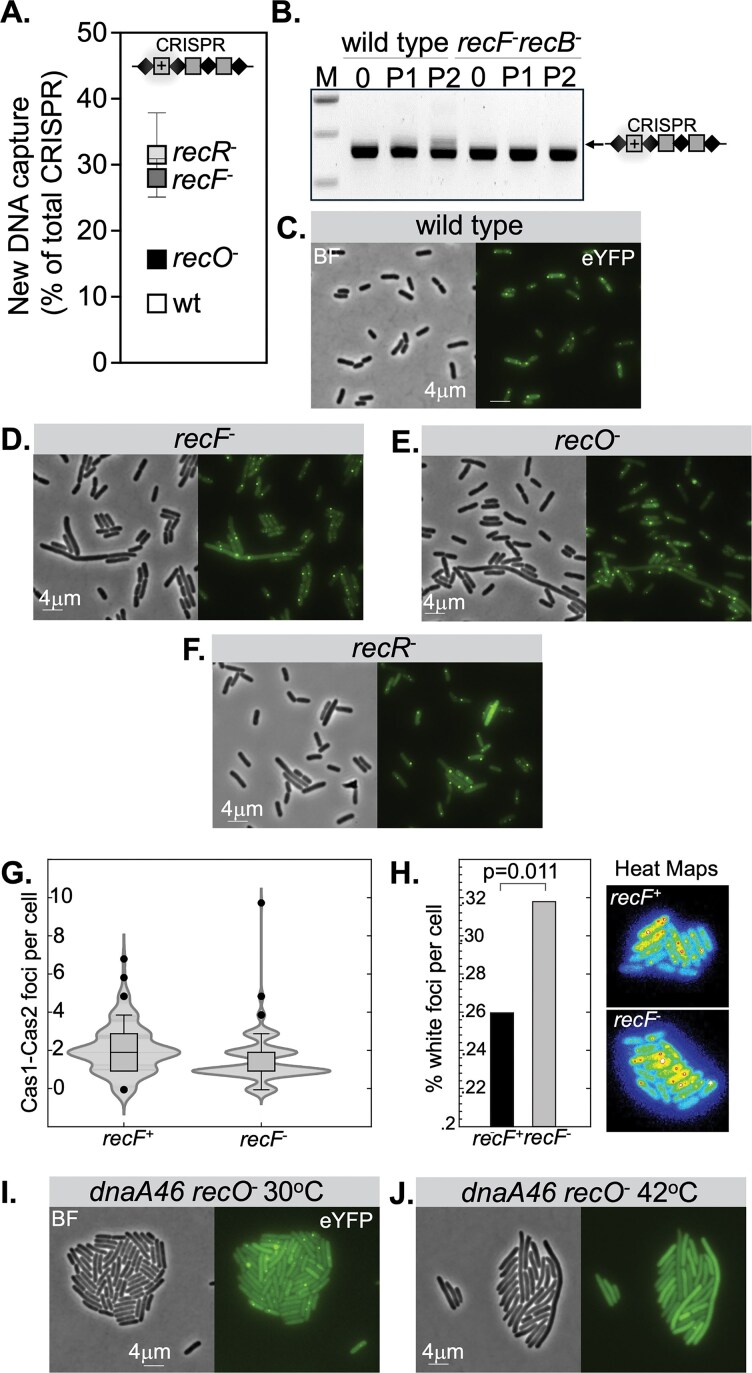
Attenuated DNA gap repair by RecFOR stimulates Cas1–Cas2**. (A**) *Escherichia coli* cells lacking any one of the RecF, RecO, or RecR proteins show increased Cas1–Cas2-catalysed spacer acquisition. The graph shows spacer acquisition measured from three independent tests, with standard deviation shown, for the first passage of cell growth (P1). Representative gel images for each are in Supplementary Fig. S9. (**B**) Representative gel comparing spacer acquisition in wild-type cells with loss of spacer acquisition in cells lacking both RecFOR (*recF*^−^) and RecBCD (*recB*^−^). (**C**–**F**) Each microscopy panel shows brightfield (BF, left) and fluorescent (eYFP, right) images. Cas1–Cas2 foci in wild-type cells are contrasted with clustered and hyper-bright Cas1–Cas2 foci in cells lacking any one of RecF, RecO, or RecR, as indicated. (**G**) Distribution of foci numbers in *recF*  ^–^ cells compared with wild-type cells, in each case from 750 cells in total. The box inside the violin plot spans 75% of the data points and shows the median point as a line. (**H**) Plots of focus brightness for wild-type cells (*recF*^+^) and cells lacking RecF (*recF*^−^), measured from 750 cells in each case. The plots show statistically significant increased brightness of foci in *recF*^−^ cells, measured and calculated as described in detail in the ‘Materials and methods’ section. (**I, J**): Hyper-bright Cas1–Cas2 foci in *recO*^−^  *dnaA*46 cells that can initiate replication at permissive temperature (30°C) disappear when they become unable to initiate replication at 42°C.

Cas1–Cas2 captures DNA during active genome duplication [10, 11] and integrates the DNA into CRISPR loci. Deleting the CRISPR locus of *E. coli* cells had no effect on Cas1–Cas2 focus formation (Supplementary Fig. S7A), indicating that foci represent Cas1–Cas2 responding to replication-dependent DNA capture, rather than binding to a CRISPR locus during DNA integration. To identify how Cas1–Cas2 interacts with DNA during replication more precisely, we compared the behaviour and activities of Cas1–Cas2 in normal cells, cells undergoing replication stress induced by replisome mutations (Fig. 3) or UV light (Fig. 4), and cells defective at post-replication DNA gap repair (Fig. 5).

Each mutation we used to attenuate replisome helicase or polymerase functions induces protein instability, when cells are shifted from permissive temperature (30°C) to non-permissive (42°C). This triggers replication stress as the proteins, and therefore the replisome, falter. As a control, we firstly tested whether Cas1–Cas2 foci were in any way affected in normal cells by this temperature shift and observed that there was no effect (Supplementary Fig. S7B). We then imaged the Cas1–Cas2 foci in cells carrying the *dnaC7* allele [54], which encodes a variant DnaC protein. DnaC ordinarily loads the DnaB helicase on to DNA and then dissociates from it. DnaB then hydrolyses ATP, translocates DNA, and separates parental DNA strands for new DNA synthesis. However, prior work has indicated that at 42°C DnaC7 remains undissociated, inhibiting DnaB at translocation and therefore halting genome duplication [54, 55]. In *dnaC7* cells at 30°C, Cas1–Cas2 foci appeared normal but they became rare in cells at 42°C (Fig. 3B). This is direct evidence for Cas1–Cas2 binding to DNA only when replisomes are advancing and synthesizing DNA, and fits with much reduced DNA capture by Cas1–Cas2 cells with a different *dnaC* allele (*dnaC2*), which cannot initiate DNA synthesis [10].

Changes to Cas1–Cas2 foci were especially striking in cells carrying the *dnaE486* allele [56], which encodes a DNA polymerase III alpha subunit with the mutation Ser-885-Pro, rendering it unstable at non-permissive temperature (42°C). At 42°C, DNA synthesis in these cells is impeded but the DnaB helicase continues to advance, accumulating DNA gaps on the leading strand and uncoupled lagging strand DNA synthesis that triggers the SOS response and repair of the gaps by recombination [57, 58]. At 30°C, Cas1–Cas2 foci were apparent in cells with normal cellular morphology but at 42°C showed hyper-bright Cas1–Cas2 foci in clusters after 120 min, in elongated cells in the SOS state (Fig. 3C). Most significantly, when substituting Cas1–Cas2 for Cas1^R84G^–Cas2, which cannot bind to and capture DNA into CRISPR sites, we saw no Cas1–Cas2 foci at all in the elongated *dnaE486* cells at 42°C (Fig. 3D).

Therefore, Cas1–Cas2 bound to DNA formed during active genome duplication (*dnaC7* data) exacerbated by replication stress (*dnaE486* data). To test whether the target could be replication fork DNA within replisomes, we co-expressed Cas1-eYFP–Cas2 with DnaQ-mCherry, the latter identifying the position of replisomal DNA polymerase III. Some cells showed co-incident mCherry and eYFP foci but these were infrequent compared with cells showing clear separation of the foci (Fig. 3E). Although the images are not amenable to systematic statistical analysis due to the very low mCherry signal, the fact that there is a large proportion of cells with clear space between foci supports that Cas1–Cas2 is targeting DNA in the vicinity of advancing replication forks, not forks themselves.

### Synchronous Cas1–Cas2 DNA binding and activated replisomes after UV-induced replication stress

UV irradiation triggers accumulation of DNA gaps and ceases replisomal DNA synthesis for at least 30 min, as global DNA damage is repaired [21, 22, 43, 59–62]. UV irradiation should therefore also change Cas1–Cas2 dynamics, if Cas1–Cas2 DNA binding responds to active replisomes. To test this, cells in early exponential phase were irradiated with 30 J/m^2^ of UV light, a dose causing >1000 lesions per chromosome [43], or mock treated with white light. After 60 min of subsequent growth, UV-treated cells were filamentous, undergoing DNA damage repair within the SOS-response (Fig. 4A). At 60 min, only 24% UV-treated cells contained Cas1–Cas2 foci, compared with 67% mock-treated cells (for each treatment *n* = 800 cells) (Fig. 4A and B). However, at 90 min post-UV treatment, Cas1–Cas2 foci were present in 90% cells, and often clustered (Fig. 4A and B). In contrast to Cas1–Cas2, replisome (YPet-DnaN) foci were abundant at both 60- and 90-min after UV treatment (Fig. 4C).

This difference between Cas1–Cas2 foci and replisomes at 60 min may represent halted but intact replisomes, and therefore a lack of DNA targets for Cas1–Cas2 when genome duplication stops. To investigate this, we used fluorescence microscopy to directly visualize positions of replication origins and termini via FROS (fluorescent repressor-operator system [63, 64]). This placed a lacO240 DNA array 16 kb from *oriC* and a tetO240 array 50 kb from the *dif* site in the termination region of the chromosome (Fig. 4D). By co-expression of LacI-mCherry, which binds lacO240, and TetR-mCerulean, which binds tetO240, alongside Cas1-eYFP-Cas2, we visualized foci in mock-treated and UV-irradiated cells. As observed before [43], 60 min after UV treatment the LacI foci were brightly compacted in cells, compared with mock treatment, but TetR foci were not. This result is in line with the assumption that genome duplication continues to initiate at *oriC*, while ongoing forks do not proceed to the terminus region [65], and that sister chromatids re-aligned to facilitate homologous exchanges for damage repair [66]. As before (Fig. 4A), Cas1–Cas2 foci were few in number at 60 min, compared with mock-treated cells (Fig. 4E). At 90-min post-UV, numerous LacI and TetR foci are observed, which are spatially separated (Fig. 4F), a pattern continuing for at least 120 min (Supplementary Fig. S8), highlighting that multiple rounds of DNA synthesis are now rapidly generating multiple copies of the chromosome [43]. This large-scale synthesis is associated with a high number of Cas1–Cas2 foci (Fig. 4F). Taken together, our data support that Cas1–Cas2 binds DNA—and therefore forms foci—only when replisomes are advancing. Taken with the data from conditional replisome mutations and UV light, and the known DNA binding proclivities of Cas1–Cas2, it strongly points to targeting of DNA gaps arising during the repair of normal and stressed genome duplication. These DNA gaps are predominately repaired by homologous recombination [24], therefore we tested the effect of attenuating this type of DNA repair on DNA capture by Cas1–Cas2.

### Defective RecFOR-mediated DNA gap repair hyper-activates Cas1–Cas2

We know that Cas1–Cas2 targets ssDNA and partial/tailed duplexes are targeted by Cas1–Cas2 and that these are not located at replication forks, but are close by, and therefore could be post-replication DNA gaps. We therefore reasoned that DNA capture by Cas1–Cas2 may be stimulated in cells with defective gap repair, when the preferred DNA capture substrate for Cas1–Cas2 persists. To test this, we attenuated RecFOR and RecBCD complexes, which set up RecA for DNA gap repair by recombination [20, 26, 30, 31, 37–40, 67, 68]. It is already known that the RecBCD helicase-nuclease assists DNA capture by Cas1–Cas2—loss of RecBCD greatly depletes it [10, 12, 13, 33]. Disrupting RecFOR had the opposite effect—deletion of any one of RecO (*recO*^−^), RecF (*recF*^−^), or RecR (*recR*^−^) stimulated DNA capture by Cas1–Cas2, compared with wild-type cells (Fig. 5A and Supplementary Fig. S9). MiSeq reads of CRISPR spacer sequences confirmed this, showing two-fold more spacers in the CRISPR locus of *recF*^−^ cells, compared with wild-type cells (respectively, 7.02 million and 3.35 million spacer reads, Supplementary Table S1). And, interestingly, cells lacking both RecF and RecB (*recF*^−^  *recB*^−^) gave no detectable naïve adaptation (Fig. 5B), highlighting that the stimulatory effect of *recF*^−^ on Cas1–Cas2 relies on RecBCD. We were unable to use RecF (or RecO) encoding genes or complementation tests that should reverse the effect of *recF*^−^ on Cas1–Cas2 DNA capture, because plasmid expression of RecF protein is highly toxic [31].

Changed Cas1–Cas2 foci were apparent in *recO*^−^, *recF*^−^, and *recR*^−^ cells, compared with wild-type cells (Fig. 5C–F). Total numbers of foci were mildly reduced (Fig. 5G), but foci appeared that were extraordinarily bright. To quantify this brightness, we converted fluorescent images into a heat map and counted the number of cells with white/saturated foci in both wild-type and *recF^–^* cells. From 750 cells analysed per strain, we observed a significant increase in the *recF^–^* cells (Fig. 5H). These hyper-bright foci were dependent on replication-dependent DNA gap repair, because they disappeared when DNA replication was not permitted in cells, because of conditional mutations in the replisome (Fig. 5J). Therefore, DNA structures targeted by Cas1–Cas2 are more prevalent and/or more readily accessible for capture by Cas1–Cas2 when RecFOR is absent from post-replication gap repair. This strongly indicates that Cas1–Cas2 builds CRISPR immunity *de novo* by capturing single-stranded or partially duplex DNA that is formed as an inevitable by-product of genome duplication, including host-dependent replication of invader MGEs.

## Discussion

Changes in cellular dynamics of Cas1–Cas2 complexes and proficiency of DNA capture into a CRISPR locus revealed replicative DNA gap repair intermediates as a likely source of new spacers that generate CRISPR–Cas immunity *de novo* (naïve adaptation). Cas1–Cas2 foci in *E. coli* required Cas1–Cas2 to be able to bind to DNA, appeared only when genome duplication was active, and were increased in response to changes in DNA replication and repair. Non-functional Cas1–Cas2 mutant complexes were unable to form these foci, corresponding with their inability to capture DNA. Several lines of evidence support that the foci represent active Cas1–Cas2 complexes, including that focus-forming complexes (Cas1-eYFP-Cas2) capture and integrate DNA into the CRISPR locus when there is no other source of Cas1–Cas2 in cells, and that the DNA-binding defective Cas1^R84G^–Cas2 did not form foci when DNA replication was activated.

We propose that Cas1–Cas2 targeting of replicative DNA intermediates on the *E. coli* chromosome—as observed in experimental naïve adaptation assays—can be extrapolated to targeting of MGE DNA that is being replicated in abundance, by the same host replisomes and DNA repair proteins. This model removes the need for Cas1–Cas2 to rely on specialized structures or sequences for distinguishing MGE DNA from ‘self’ DNA: during infection, MGE DNA is amplified as tens of genome copies [69–71], generating abundant ssDNA gap intermediates via semi-conservative, concatemeric, or rolling circle modes of replication [72, 73]. These intermediates are targets for Cas1–Cas2, assisted by RecBCD, which is recruited to replicative DNA repair sites. Consistent with this, UV irradiation of bacteriophage prior to infection—which damages bacteriophage DNA such that subsequent replication requires more gap repair—is known to greatly stimulate Cas1–Cas2 DNA capture [74]. PAM sequences preferred by Cas1–Cas2 active sites, which would be present at abundant gap repair sites, subsequently allow avoidance of self-targeting by interference effector complexes once immunity has been established by Cas1–Cas2, aided by RecBCD. DNA gap repair intermediates—ssDNA flanked by duplex DNA with exposed 3′and 5′ends, as ‘tailed’ or ‘partial’ duplexes—are well-suited substrates for Cas1–Cas2, based on their binding with high affinity by Cas1–Cas2 *in vitro*, and evidence recovering ssDNA from Cas1–Cas2 in cells, pre-integration into CRISPR [13, 15, 16]. Cas1–Cas2 is an effective nuclease across bacteria and archaea, which can excise DNA branches apparently without assistance from other nucleases [13, 14, 17, 18], although multiple studies make clear that the RecBCD nuclease-helicase is required for Cas1–Cas2 to capture and integrate DNA *de novo*.

Our primary aim was to identify how Cas1–Cas2 is licensed by DNA replication and DNA repair to generate immunity in the absence of guidance from interference effectors. The strong stimulatory effect of deleting RecFOR was unexpected and strongly implicates DNA gap repair, while preserving the well-known dependence of Cas1–Cas2 on RecBCD in cells [10, 12, 13, 33]. RecBCD and RecFOR protein complexes repair replicative DNA breaks and gaps in bacteria [37–40], where they have distinct roles, as evidenced by low viability of *recF*^−^  *recB*^−^ cells after UV irradiation, compared with either single mutant [39]. Because RecFOR and Cas1–Cas2 both prefer ssDNA and ssDNA-tailed duplexes [15, 30, 31], deletion of RecFOR would present Cas1–Cas2 with more accessible DNA for capture. RecFOR stabilizes stalled replication forks and protects them from nucleolytic degradation by RecJ [44, 75], loading RecA at these sites for recombination. However, it is unlikely that the stimulatory effect of RecFOR deletion acts through increased RecJ nuclease activity: that scenario would not require RecBCD, and mononucleotide products of RecJ [76] are unsuitable for capture by Cas1–Cas2.

RecBCD is a DNA translocase and nuclease that resects ssDNA ends at DNA strand breaks, from which it loads RecA to initiate recombination [77]. It is notable that Cas1 physically interacts with RecB and RecC [14]; this interaction may form a sub-complex protecting ssDNA from nuclease activity at gap repair sites [78]. Accordingly, in cells expressing Cas proteins in response to an MGE, a RecBC-Cas1-Cas2 interaction could be mobilized to replicating MGE DNA at sites requiring repair, facilitating DNA capture prior to recombination by RecA—consistent with naïve adaptation being independent of RecA [12]. This RecBC-Cas1–Cas2 interaction would also explain why RecB is required for DNA capture by Cas1–Cas2 even when RecFOR was absent: RecBC would enable access to tailed and partial duplex DNA otherwise protected by SSB. Moreover, RecFOR sub-complexes—primarily RecFR and RecOR—bind to DNA gaps to load RecA and trigger recombination; we suggest this activity is antagonized by RecBC(D) in cells, which directs Cas1–Cas2 to DNA intermediates when cells are under attack from replicating MGEs.

The finding that Cas1–Cas2 foci were not dependent on a CRISPR DNA site demonstrates that foci do not report spacer integration reactions, even though foci-forming Cas1-eYFP-Cas2 complexes are proficient at spacer integration. We were, however, unable to directly demonstrate the transit of a single Cas1–Cas2-DNA complex from a focus—that we propose marks a gap repair site—to a CRISPR locus, where it would generate a new spacer. Doing so would require single molecule imaging of Cas1–Cas2 within live cells, which is beyond the scope of this study, and has so far not been achieved. We estimate at least 50 Cas1–Cas2 complexes per focus, based on comparing focus intensities with those of *E. coli* DnaQ and the β2 clamp [79, 80]. DNA gaps arise during normal genome duplication and increase in frequency under replication stress, such as caused by UV irradiation [21–23]. Measurements using fluorescent SSB and replisome proteins suggest 5–11 ssDNA gap regions are associated with each advancing replication fork under normal growth conditions, comprising 0.5–1.0 kb of DNA [80–82]. This is consistent with the very bright Cas1–Cas2 foci observed during replication stress (e.g. following UV irradiation or loss of RecFOR), and the fewer but clearly visible foci that accumulate during normal replication at ssDNA gap regions, given that the Cas1–Cas2 footprint is about 30 nucleotides.

It is likely that Cas1–Cas2 would target DNA gap intermediates only during MGE invasion, rather than during unperturbed genome duplication of a healthy host cell, because MGE invasion triggers the LeuO protein to de-repress *cas* gene expression, which is otherwise silenced by the H-NS transcription factor [83–85]. DNA capture assays, as used in this work, typically use inducible plasmid expressed Cas1–Cas2, which is free from H-NS repression and therefore targets chromosomal DNA gap intermediates during host genome duplication. This ‘auto-immunity’ represents a limitation of such experimental systems, but has nonetheless provided understanding of Cas1–Cas2 molecular mechanism [4–6], and it’s interplay with DNA replication and repair [10–13]. In this work, visualizing responses of Cas1–Cas2 to genome duplication and replicative DNA gap repair has revealed how MGE replication—enabled by host proteins—licenses Cas1–Cas2 to generate CRISPR immunity *de novo* from gap repair intermediates. This behaviour of Cas1–Cas2 likely extend beyond *E. coli*, given that replicative DNA gap repair is essential across prokaryotes and eukaryotes [26, 86–89]. Furthermore, the behaviour of Cas1–Cas2 foci has potential as an inducible, plasmid-encoded biomarker for replication stress responses and replisome activity in human and yeast cells, offering a tool to investigate the genetic factors that control them.

## Supplementary Material

gkag564_Supplemental_File

## Data Availability

The data referred to in this work have been deposited in Figshare (https://figshare.com) with digital object identifier: 10.6084/m9.figshare.30813251
