# Supplementary material for: Visualizing the interplay of Cas1–Cas2 with DNA replication-repair that creates CRISPR–Cas immunity
Source: Nucleic Acids Res. 2026 Jun 8;54(11):gkag564. doi: 10.1093/nar/gkag564 (PMC13244154; doi:10.1093/nar/gkag564)
Supplement: gkag564_Supplemental_File [file gkag564_supplemental_file.docx]

**Supplementary Data & Information**

**Figure S1:** SDS-PAGE showing 1 μg each of purified Cas1 (lane 1), Cas1-eYFP (lane 2) and Cas1^R84G^-eYFP (lane 3). The protein marker lane is taken from the same gel with irrelevant lanes omitted in between.

**
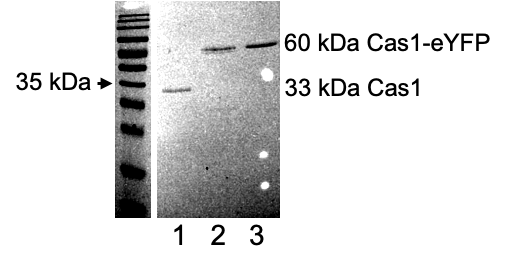
**

**Figure S2: A.** Representative gel showing that combining expression of fused Cas1-eYFP with fused Cas2-mCherry is proficient at naïve adaptation in *E. coli* cells, but that fused Cas1-mCherry with Cas2-eYFP is not. The expanded CRISPR-1 locus of the 2^nd^ passage (P2) of the former is arrowed, with no detectable expansion for the latter. **B.** Aberrant foci, at cell poles, formed from expressing an inactive prototype Cas1-eYFP-Cas2 that was unable to catalyse DNA capture into CRISPR loci in cells.


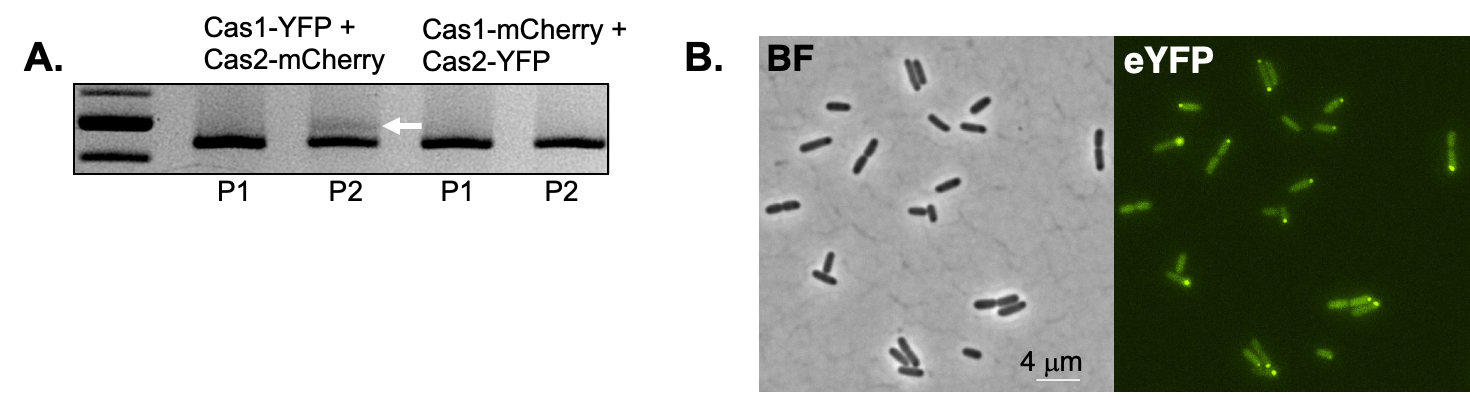


**Figure S3:** SDS-PAGE gel (12.5% acrylamide) of purified Cas1 and Cas1^V76L^ proteins (10μg) used in *in vitro* DNA binding assays.


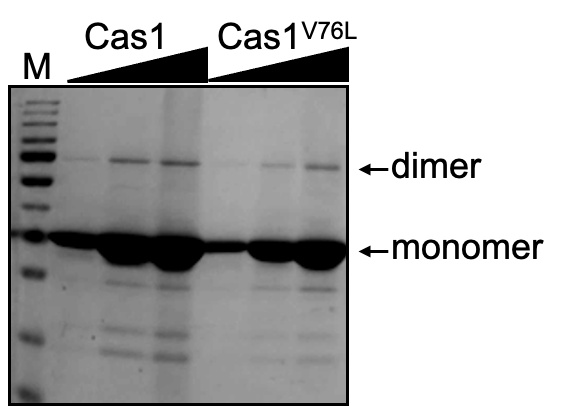


**Figure S4:** DNA anisotropy measurements showing binding of Cas1 when mixed with a 5’ single strand tailed DNA duplex, a 3’ tailed duplex, or a fully base-paired duplex (each 40 nM). Each data set was in duplicate, and Cas1 was used at concentrations (nM) of 62.5, 93.75, 125,187.5, 250, 375, 500, 750 and 1000. Reactions were incubated for 5 minutes (5’) or 10 (10’) minutes, as indicated before taking the measurements. Error bars are standard error from the mean.

**
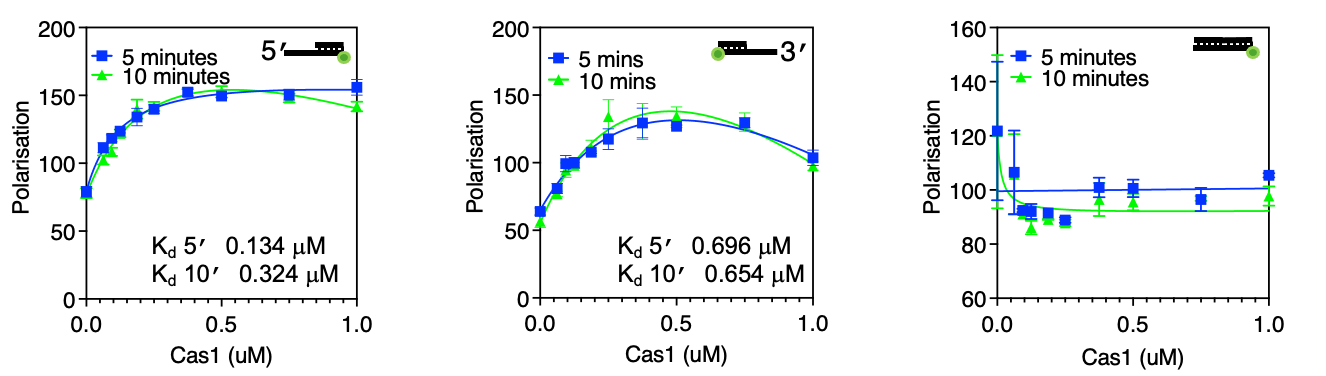
**

**Figure S5:** Images from AlphaFold Multimer docking Cas1-Cas2 with *E. coli* DnaK, two proteins that are known to physically interact but at unknown binding sites (1).

A: DnaK is shown green, and Cas1 orange, with the Val-76 containing beta-sheet fold highlighted in black.

B: Zoom in of the predicted DnaK-Cas1 interaction region, using the same colours as in A, but with addition of purple space-fill Valine-76 residues.

**
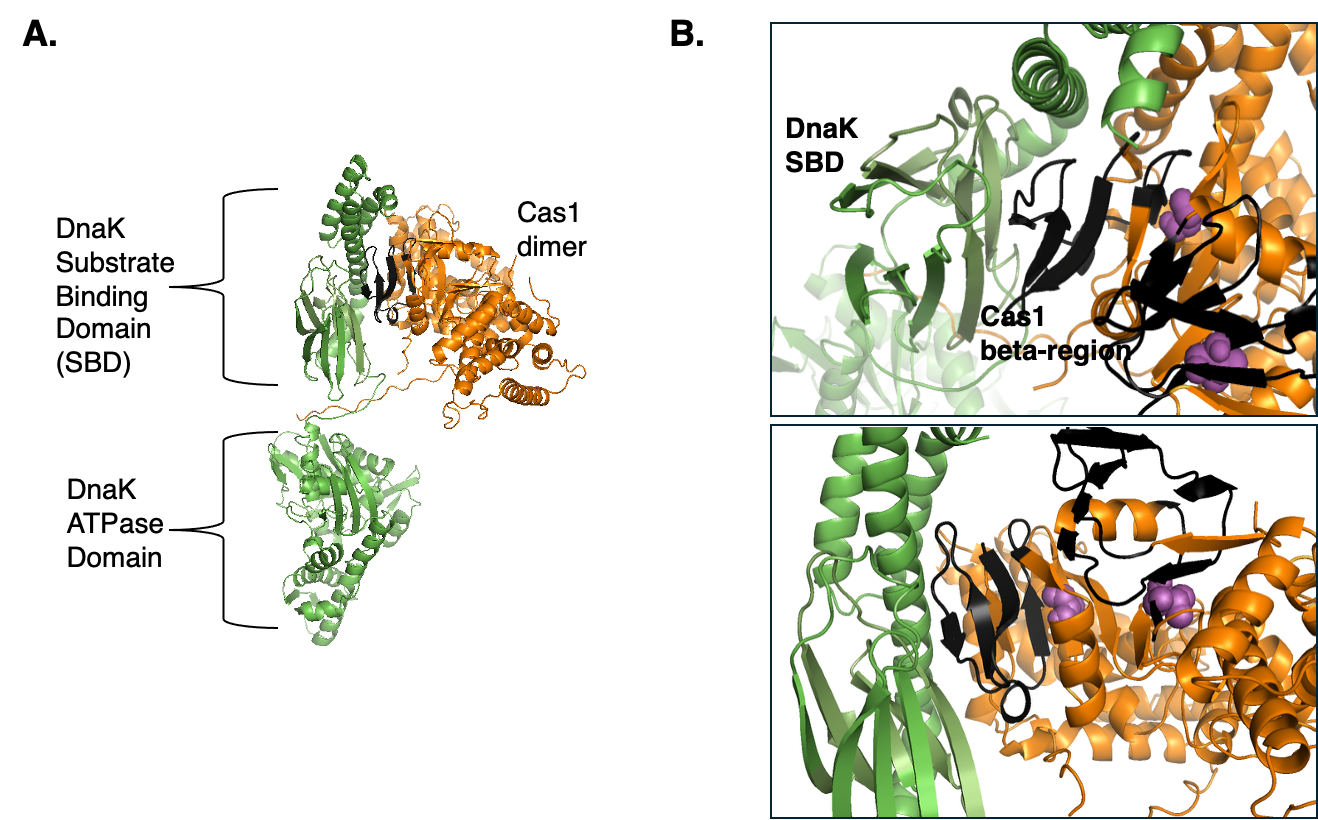
**

**Figure S6:** Hyper-abundant NmVenus-Cas1^V76L^ protein (lane 4) as indicated, compared with undiscernible wild type NmVenus-Cas1 (lane 2), and NmVenus-Cas1^D218A^ (lane 3). Lane 1 shows plasmid expression of Cas1 alone, without fusion to mVenus. The panel shows a Brilliant Blue stained SDS-PAGE gel.


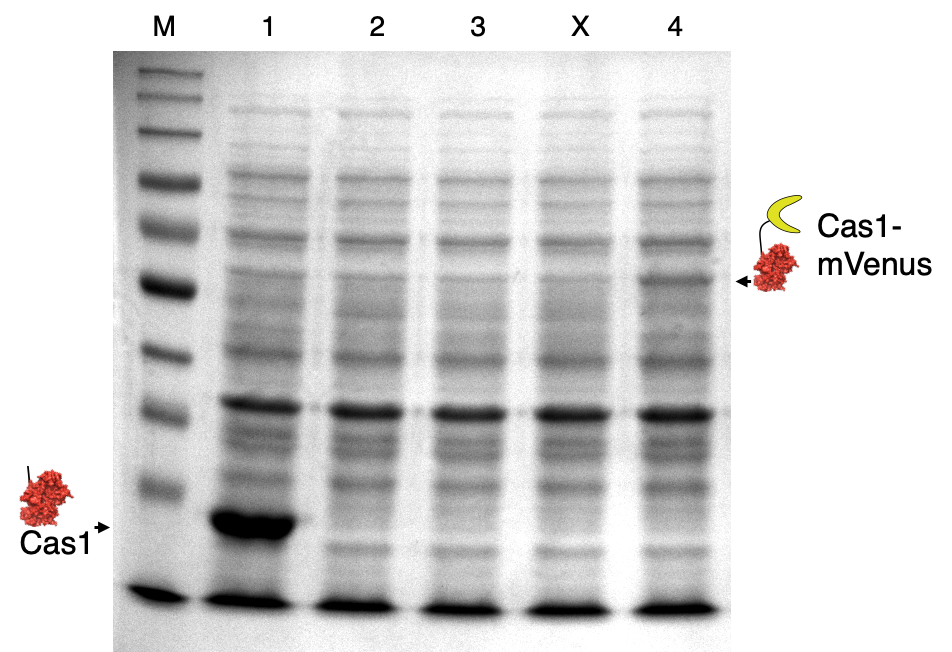


**Figure S7:** Control experiments showing (A). that deletion of the chromosomal CRISPR site for DNA capture in *E. coli* cells has no apparent effect on Cas1-Cas2 focus formation, and (B). that growing *E. coli* cells at 42^o^C has no apparent effect on normal foci formation by Cas1-Cas2, or aberrant rare polar foci of Cas1^R84G^-Cas2.

**
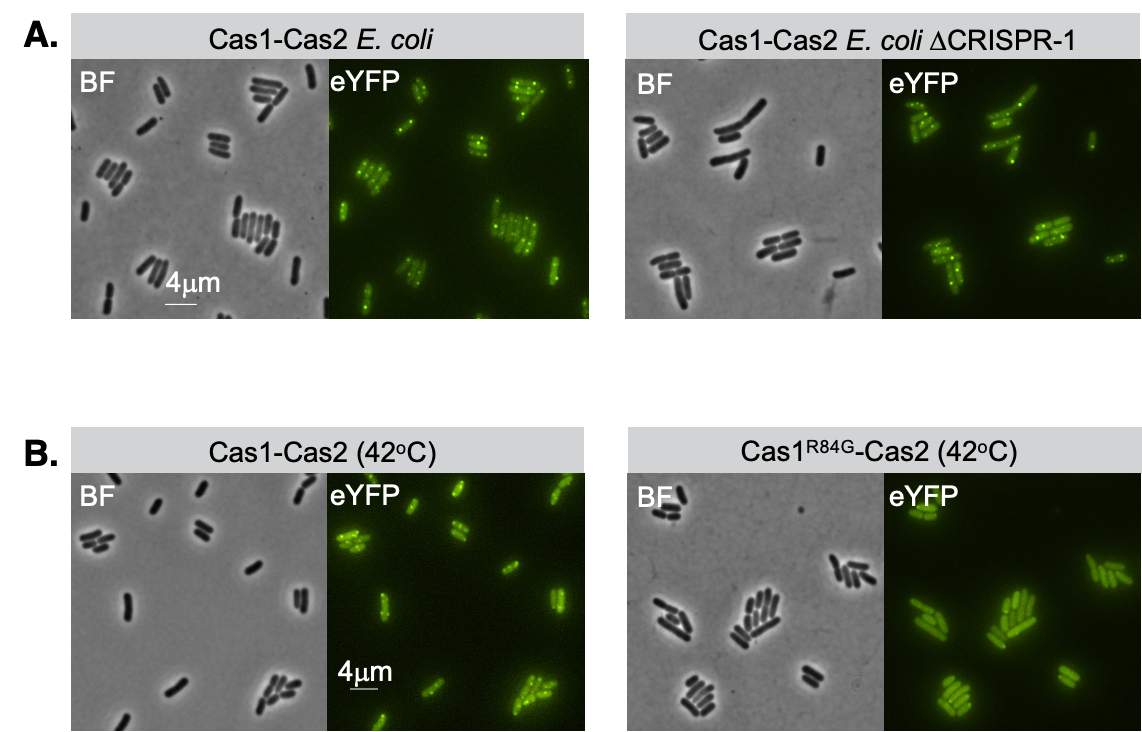
**

**Figure S8:** See also Figure 6 for full details − these images are for the 120-minute time point in which Cas1-Cas2 and replisome foci are very similar to at the 90-minute time point.


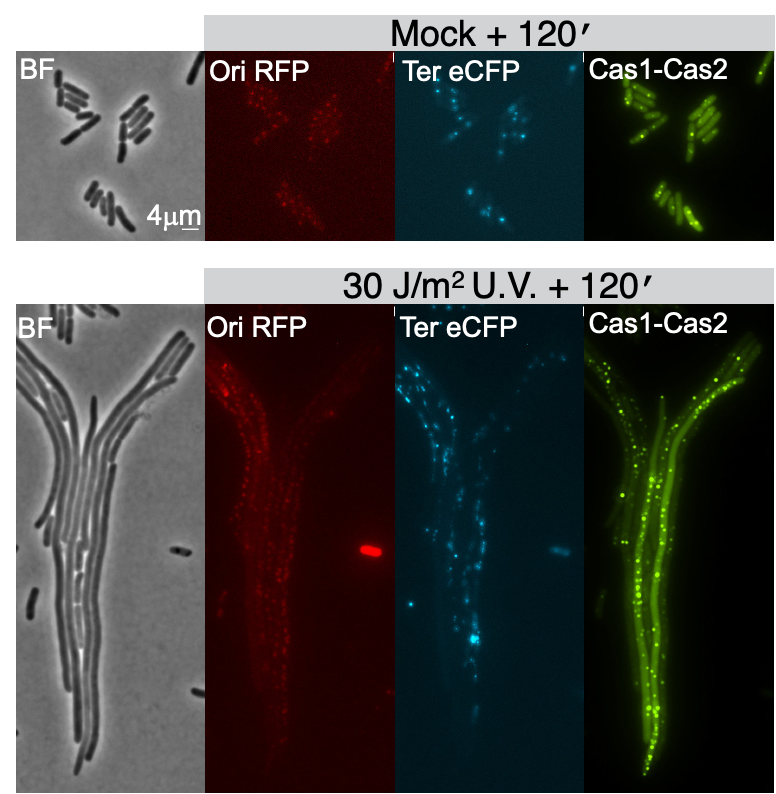


**Figure S9:** Representative gels for data in Figure 5A. They illustrate enhanced spacer acquisition into the CRISPR-1 locus in any of the *recF, recO* or *recR* deletions, detected by PCR.


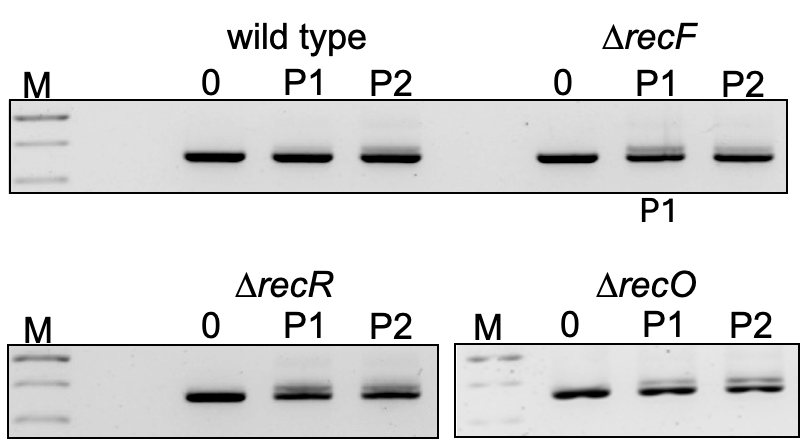


**SUPPLEMENTARY TABLES**

**Table S1: Spacer reads of wild type and *recF*^-^ strains**

| **Strain DNA** | **Spacer reads (filtered: 31–33 bp)** | **Map to Cas1-Cas2 plasmid** | **Unique to E. coli reference genome NC_000913.2** |
| --- | --- | --- | --- |
| WT (IIB1165) | 3,351,089 (3,343,454) | 1,944,114 | 1,282,290 |
| *recF* ^-^(IIB1467) | 7,022,624 (7,005,281) | 5,092,558 | 1,715,214 |

**Table S2: *E. coli* strains**

| **Strain** | **Relevant Genotype** | **Reference/Source** |
| --- | --- | --- |
| BL21 A.I. | *araB*::T7RNAP*-tetA* | Invitrogen |
| MG1655 | F^–^ rph-1 | (2) |
| AB1157 | F^-^ *thr-1 leuB6* Δ*(gpt-proA)62 hisG4 thi-1 argE3 lacY1 galK2 ara-14 xyl-5 mtl-1 tsx-33 supE44 rpsL31 kdgK51 rfbD1 mgl-51* λ^-^ *rac^-^* | (3) |
| BW25113 | F^-^ *rrnB* Δl*acZ4748*(::*rrnB-3*) *hsdR514* Δ(*araBAD*)*567* Δ(*rhaBAD*)*568* *rph-1* λ^-^ | (4) |
| **MG1655 Derivatives** | | |
| EB377 | araB::T7RNAP-*tetA* | ELB collection |
| EB393 | *dnaE486 zae-502*::Tn*10* | (5) |
| AM1764 | *ΔrecO::kan* | (6) |
| AS1062 | *<kan>-ypet-dnaN* | MG1655 × P1.RRL190 to Km^r^ |
| JD1031 | *ΔrecG*::*apra ΔrecO*::*kan* | RCe452 × P1.N6619 to Km^r^ |
| JD1227 | *dnaQ-mTagRFP-kan* | MG1655 × P1.RRL546 to Km^r^ |
| JD1708**^b^** | MG1655 pTK135 (Cas1eYFP-Cas2) | MG1655 × pTK135 to Ap^r^ |
| JD1772 | MG1655 pTK148 (Cas1eYFP-mCherry-li-Cas2) | MG1655 × pTK148 to Ap^r^ |
| JD1777 | *dnaC7* pTK135 (Cas1eYFP-Cas2) | RCe079 × pTK135 to Ap^r^ |
| JD1814 | *recR1*::*EZdhfr* | MG1655 × P1.AU1102 to Tm^r^ |
| JD1815 | *ΔrecO*::*kan* | MG1655 × P1.JD1031 to Km^r^ |
| JD1816 | *recR1*::*EZdhfr* pTK135 (Cas1eYFP-Cas2) | JD1814 × pTK135 to Ap^r^ |
| JD1817 | *ΔrecO*::*kan* pTK135 (Cas1eYFP-Cas2) | JD1815 × pTK135 to Ap^r^ |
| JD1818 | *tnaA*::Tn*10 recF143* pTK135 (Cas1eYFP-Cas2) | JD1254 × pTK135 to Ap^r^ |
| JD1836 | MG1655 pTK185 (Cas1^D218A^eYFP) | MG1655 × pTK185 to Ap^r^ |
| JD1839 | *dnaQ-mTagRFP-kan* pTK135 (Cas1-li-eYFP-Cas2) | JD1227 × pTK135 to Ap^r^ |
| JD1841 | RRL189 (see below) x pTK185 | This work |
| JD1857 | MG1655 pTK194 (Cas1^V76L^eYFP) | This work |
| N6619 | *ΔrecO*::*kan* | MG1655 × P1.AM1764 to Km^r^ |
| N6822 | *ΔlacIZYA Δrep*::*cat dnaC↑ zji*::Tn*10 ΔpriA*::*apra recR1*::*dhfr* | R.G. Lloyd, unpublished |
| RCe079 | *dnaC7* | (7) |
| RCe692 | *ΔrecO*::*kan dnaA46 tnaA*::Tn*10* | N6619 × P1.AU1054 to Tc^r^ |
| RCe854 | MG1655 pTK203 (Cas1eYFP) | MG1655 × pTK203 to Ap^r^ |
| RCe861 | MG1655 pTK206 (Cas1^R252E^eYFP) | MG1655 × pTK206 to Ap^r^ |
| RCe904 | *dnaE486 zae-502*::Tn*10* pTK135 (Cas1eYFP-Cas2) | EB393 × pTK135 to Ap^r^ |
| RCe922 | *dnaE486 zae-502*::Tn*10* pTK136 (Cas1^R84G^eYFP-Cas2) | EB393 × pTK135 to Ap^r^ |
| **BW25113 derivatives** | | |
| IIB1165 | +Δ*cas3*::*apra* Δ(*casC-cas1*::*FRT*) | (8) |
| IIB1465 | IIB1165, + Δ*recF735*::*kan* | IIB1165 x P1. IIB1463 |
| IIB1487 | IIB1165, + Δ*recR776*::*kan* | IIB1165 x P1. IIB1484 |
| IIB1521 | IIB1165, + *recO1504*::Tn*5* | IIB1165 x P1. IRB103 |
| IIB1463 | Δ*recF735*::*kan* | This work |
| IIB1467 | Δ*recF735*::*FRT* | This work |
| IIB1484 | ΔrecR776::kan | This work |
| IIB1494 | As IIB1165, + Δ*recF735*::*FRT* Δ*recB745*::*kan* | IIB1467 x P1. JW2788-1 |
| IIB1549 | As IIB1165, +ΔCRISPR-1::kan | This work |
| **AB1157 derivatives** | | |
| RRL546 | *dnaQ-mTagRFP-kan* | (9) |
| RRL189 | *lacO240::hyg* at *ori1*^a^, *tetO240::gen* at *ter3*^a^, *Plac-lacI-mCherry-frt* at *leuB*, *Plac-tetR-mCerulean-frt* at *galK*. | (9) |
| RRL190 | *<kan>-ypet-dnaN* | (10) |
| IRB103 | *recO1504*::*Tn5* | IIB collection, unpublished |

**a −** Only the relevant additional genotype of the derivatives is shown. The abbreviations ‘kan’, ‘apra’, ‘dhfr’, ‘cat’, ‘tet’ and ‘Tn*10*’ refer to insertions conferring resistance kanamycin (Km^r^), apramycin (Apra^r^), trimethoprim (Tm^r^), chloramphenicol (Cm^r^) and tetracyclin (Tc^r^). ‘<>’ indicates the use of *frt* sites, where *frt* stands for the 34 bp recognition site of the FLP/*frt* site-directed recombination system. Thus, *<kan>* refers to a chloramphenicol marker flanked by an *frt* site either side. If the *frt* site was removed via Flp recombinase, this is shown by ‘<>’, indicating that a single *frt* scar is left in the chromosome.

**b –** For all fluorescently tagged proteins, the tagged protein and the fluorescent protein are separated by a (GGS)_8_ linker, indicated by ‘li’, as was previously described (1). For all plasmids with mutant versions of either Cas1 or Cas2 we indicate the mutation in square brackets following the mutated protein. For further details on plasmids used in this study see Supplementary Table S3.

**DNA binding substrates for EMSAs and fluorescent anisotropy** were formed from combinations of the following oligonucleotides, annealed together and gel purified to remove unannealed ssDNA, to generate either 3’or 5’tailed partial duplexes, or the fully base-paired duplex. Use of either the Cy5 or FAM tagged oligo is as in the Results:

FAM DNA:FAM-5’AATCAAAGTGGACCCAACTCGAAATCAACCGTAACAACAAGCAGGC

Cy5-DNA: Cy5-5’AATCAAAGTGGACCCAACTCGAAATCAACCGTAACAACAAGCAGGC

DNA-12A: 5’GCCTGCTTGTTG

DNA-12B: 5’TCCACTTTGATT

DNA46: 5’GCCTGCTTGTTGTTACGGTTGATTTCGAGTTGGGTCCACTTTGATT

**Table S3:** DNA primers for gene recombineering (deletions), molecular cloning and site-directed mutagenesis

| **Primer** | Sequence (5’to 3’) |
| --- | --- |
| recFdelF | GCGGCCAGCCAGAGCGCGGCTTATGTTGTCATGCCAATGAGACTGTAATGATTCCGGGGATCCGTCGACC |
| recFdelR | TCAACGTTTCTCGCTCATTTATACTTGGGTTAATCCGTTATTTTACCCTTTGTAGGCTGGAGCTGCTTCG |
| recRdelF | GTATCCTCCGGAATGCAGCTGCCGCCTGGCTTTAAGATGCCGTTCTGATGATTCCGGGGATCCGTCGAC |
| recRdelR | CGAGAGCAGGTGATCCTGCTCTCGTTTGCTTAAAAACGAATCTTATGACGTGTAGGCTGGAGCTGCTTCG |
| CRISPR-1 delF | ATGGGAAAAAATGCTTTAAGAACAAATGTATACTTTTAGAATGATTCCGGGGATCCGTCGACC |
| CRISPR-1 delR | CGAGTTCCTAGTCCATCATTCCACCTATGTCTGAACTCCTGTAGGCTGGAGCTGCTTCG |
| recF-F (genotype) | CGGCTTATGTTGTCATGCCA |
| recF-R (genotype) | CGACATCAACGTTTCTCGCTC |
| recR-F (genotype) | GCGTAGAGATCGACCCGAG |
| recR-R (genotype) | AATTTCAAGCGAGAGCAGGTG |
| Cas1_V76L_F | AACATTGTTGCTGTGGGTGGGGG |
| Cas1_V76L_R | CCAACTTGCGCAGC |
| Del_Cas2_F | TCACTTGTACAGCTCGTCCATGC |
| Del_Cas2_R | CGAGATCTGCAGCTGGTACCA |
| Cas1_R252E_F | TTTGGCGTGCGAAGATATTTTTCGCAGTAG |
| Cas1_R252E_R | CGGACTTCCCGGTCC |
| GGS_linker_F | ACTACCGCCACTACCACCTGAACCACCGCTACCACCCTTGTACAGCTCGTCCATGCC |
| GGS_linker_R | GGTGGCTCAGGCGGAAGCGGTGGCAGTGGTGGCAGCTAGTAACCAACTCCATAAGGATCCGC |
| Amp135_F | AGTGGTGGCAGCAGTATGTTGGTCGTGGTC |
| Amp135_R  mCherry_GGS_F | GCCCTTGCTCACCATTTCACTTGTACAGCTC  TACAAGTGAAATGGTGAGCAAGGGCGAGGAG |
| mCherry_GGS_R | CGACCAACATACTGCTGCCACCACTGCCACC |
| Cas1_R41G_F | GACAGGGATCACTGGCCATATTC |
| Cas1_R41G_R | TTGTCGATAAGTACAAACG |
| Cas1_D218A_F | CTTTGTTTACGCTATTGCAGACATC |
| Cas1_D218A_R | GAAAGAGGCTTTCCTGTATG |
| Cas2_E9A_F | GAGGTACATTTGCAGTGACCACG |
| Cas2_E9A_R | CGCGCTTACGAGGCAGAT |
| Amp134_F | ATCACCGCCGTAAGCGGCCGCATAATGCTTAAGTC |
| Amp134_R | CCTTGCTCACGCTGCCACCACTGCCACC |
| NmV_F | TGGTGGCAGCGTGAGCAAGGGCGAGGAG |
| NmV_R | CGGCCGCTTACGGCGGTGATATAGACGTTG |

**Table S4: Plasmids**

| **Plasmid** | **Notation** | **Source/Construction/Description** |
| --- | --- | --- |
| pControlA | pBadHisA | Invitrogen™ |
| pCas1-Cas2 | pEB628 | Wild type Cas1-Cas2 expression plasmid inducible from the araBAD promoter. *E. coli ygbT* and *ygbF* cloned into pBadHisA. |
| pCas1V76L-Cas2 | pTK193 | Cas1V76L-Cas2 expression plasmid. As described for pEB628 but with enhanced adaptation Cas1V76L (11). pEB628 was amplified with primers Cas1_V76L_F/R to introduce the V76L mutation to *ygbT* pEB628 via site directed mutagenesis (SDM). |
| pCas1^eYFP^-Cas2 | pTK135 | Expresses *E. coli* Cas1 fused at the C-terminus with eYFP via a 8 × GGS linker, and Cas2. As described for pEB628, but with an 8 × GGS linker and eYFP inserted before the stop codon of *ygbT* (Cas1) (1). |
| pCas1^eYFP^ | pTK203 | Expresses Cas1 fused at the C-terminus with eYFP via a 8 × GGS linker, under control of the L-arabinose  inducible araBAD promoter. pTK135 was amplified using primers Del_Cas2 F/R to delete *ygbF* (Cas2) via site-directed mutagenesis (SDM). |
| pCas1R84G^eYFP^-Cas2 | pTK136 | As described for pTK135, but with acquisition and DNA binding deficient Cas1R84G (1). |
| pCas1R252E^eYFP^-Cas2 | pTK206 | As described for pCas1^eYFP^-Cas2, but with Cas1R252E mutant that is unable to for a complex with Cas2) (12). pTK135 was amplified using PCR primers Cas1_R252E_F/R to introduce the mutation . |
| pmCherry | #29769 | Addgene plasmid #2976. mCherry encoded in a pET vector. |
| pmCherry-8-GGS | pTK146 | 8 × GGS linker inserted at the C-terminus of mCherry in the pmCherry plasmid using primers GGS_linker_F/R |
| pCas1^eYFP^-Cas2^mCherry^ | pTK148 | Expresses *E. coli* Cas1 fused at the C-terminus with eYFP via a 8 × GGS linker, alongside *E. coli* Cas2 fused at the N-terminus with mCherry via an 8 × GGS linker, all inducible from the araBAD promoter. Whole plasmid PCR amplification of pTK135 was carried out with primers Amp135_F/R, mCherry-8-GGS was amplified with primers mCherry_GGS_F/R. PCR fragments were assembled using the NEBuilder® Hi-Fi DNA Assembly kit (New England Biolabs®). |
| pCas1D218A^eYFP^-Cas2 | pTK185 | As described for pCas1^eYFP^-Cas2, but with catalytically inactive Cas1D218A mutant (14). pTK135 was amplified using PCR primers Cas1_D218A_F/R introducing the mutation. |
| pCas1V76L^eYFP^-Cas2 | pTK194 | As described for pCas1^eYFP^-Cas2, but with Cas1 V76L enhanced adaptation mutation (11). pTK135 was amplified using PCR primers Cas1_V76L_F/R introducing the mutation via SDM. |
| pNmVenus |  | pBadHisA backbone vector containing the N-mVenus fragment inducible from the araBAD promoter (15). |
| pCmVenus |  | pRFS-1b backbone vector containing the C-mVenus fragment under control of the T7 promoter (15). |
| pCas1^BioID^-Cas2 | pTK134 | Wild type E. coli ygbT (Cas1) in frame with (GGS)_8_ linker and BioID2, alongside E. coli ygbF (Cas2) cloned into pBadHisA, inducible from the araBAD promoter (1). |
| pCas1^NmV^-Cas2 | pTK152 | Expresses Cas1 fused at the C-terminus with N-mVenus fragment via a 8 × GGS linker, alongside Cas2, under control of the of the araBAD promoter. PCR fragments were produced by amplifying pTK134 with primers Amp134_ F/R to remove BioID, and pNmVenus with primers NmV_F/R to amplify N-mVenus fragment. Fragments were assembled using the NEBuilder® Hi-Fi DNA Assembly kit (New England Biolabs®) |
| pCas1V76L^NmV^-Cas2 | pTK195 | As described for pTK152 but with Cas1 V76L enhanced adaptation mutation (11). pTK152 was amplified using primers Cas1_V76L_F/R introducing the mutation. |
| pDnaK | pTK82 | pACYCduet containing *E. coli* *dnaK* (1). |
| pDnaK^CmV^ | pTK151 | DnaK fused at N-terminus with C-mVenus under control a T7 promoter. *E. coli* *dnaK* was subcloned from pTK82 into pCmVenus using BamH1 and HinDIII. |

**SUPPLEMENTARY REFERENCES**

1. Killelea, T., Dimude, J.U., He, L., Stewart, A.L., Kemm, F.E., Radovcic, M., Ivancic-Bace, I., Rudolph, C.J. and Bolt, E.L. (2023) Cas1-Cas2 physically and functionally interacts with DnaK to modulate CRISPR Adaptation. *Nucleic Acids Res*, **51**, 6914-6926.

2. Bachmann, B.J. (1996) In Neidhardt, F. C., Curtiss III, R., Ingraham, J. L., Lin, E. C. C., Low, K. B., Magasanik, B., Reznikoff, W. S., Riley, M., Schaechter, M. and Umbarger, H. E. (eds.), *Escherichia coli and Salmonella Cellular and Molecular Biology, (Second Edition)*. ASM Press, Washington, D.C., pp. 2460-2488.

3. Bachmann, B.J. (1972) Pedigrees of some mutant strains of *Escherichia coli* K-12. *Bacteriol. Rev.*, **36**, 525-557.

4. Datsenko, K.A. and Wanner, B.L. (2000) One-step inactivation of chromosomal genes in Escherichia coli K-12 using PCR products. *Proc Natl Acad Sci U S A*, **97**, 6640-6645.

5. Guy, C.P. and Bolt, E.L. (2005) Archaeal Hel308 helicase targets replication forks in vivo and in vitro and unwinds lagging strands. *Nucleic Acids Res*, **33**, 3678-3690.

6. Rudolph, C.J., Upton, A.L. and Lloyd, R.G. (2008) Maintaining replication fork integrity in UV-irradiated Escherichia coli cells. *DNA Repair (Amst)*, **7**, 1589-1602.

7. Rudolph, C.J., Upton, A.L. and Lloyd, R.G. (2007) Replication fork stalling and cell cycle arrest in UV-irradiated Escherichia coli. *Genes Dev*, **21**, 668-681.

8. Radovcic, M., Killelea, T., Savitskaya, E., Wettstein, L., Bolt, E.L. and Ivancic-Bace, I. (2018) CRISPR-Cas adaptation in Escherichia coli requires RecBCD helicase but not nuclease activity, is independent of homologous recombination, and is antagonized by 5' ssDNA exonucleases. *Nucleic Acids Res*.

9. Reyes-Lamothe, R., Possoz, C., Danilova, O. and Sherratt, D.J. (2008) Independent positioning and action of Escherichia coli replisomes in live cells. *Cell*, **133**, 90-102.

10. Wang, X., Lesterlin, C., Reyes-Lamothe, R., Ball, G. and Sherratt, D.J. (2011) Replication and segregation of an Escherichia coli chromosome with two replication origins. *Proc Natl Acad Sci U S A*, **108**, E243-250.

11. Yosef, I., Mahata, T., Goren, M.G., Degany, O.J., Ben-Shem, A. and Qimron, U. (2023) Highly active CRISPR-adaptation proteins revealed by a robust enrichment technology. *Nucleic Acids Res*, **51**, 7552-7562.

12. Nunez, J.K., Kranzusch, P.J., Noeske, J., Wright, A.V., Davies, C.W. and Doudna, J.A. (2014) Cas1-Cas2 complex formation mediates spacer acquisition during CRISPR-Cas adaptive immunity. *Nat Struct Mol Biol*, **21**, 528-534.

13. Nunez, J.K., Harrington, L.B., Kranzusch, P.J., Engelman, A.N. and Doudna, J.A. (2015) Foreign DNA capture during CRISPR-Cas adaptive immunity. *Nature*.

14. Ivancic-Bace, I., Cass, S.D., Wearne, S.J. and Bolt, E.L. (2015) Different genome stability proteins underpin primed and naive adaptation in E. coli CRISPR-Cas immunity. *Nucleic Acids Res*, **43**, 10821-10830.

15. He, L., Lever, R., Cubbon, A., Tehseen, M., Jenkins, T., Nottingham, A.O., Horton, A., Betts, H., Fisher, M., Hamdan, S.M. *et al.* (2023) Interaction of human HelQ with DNA polymerase delta halts DNA synthesis and stimulates DNA single-strand annealing. *Nucleic Acids Res*.
